# A bacterial acetyltransferase triggers immunity in *Arabidopsis thaliana* independent of hypersensitive response

**DOI:** 10.1038/s41598-017-03704-x

**Published:** 2017-06-15

**Authors:** Jay Jayaraman, Sera Choi, Maxim Prokchorchik, Du Seok Choi, Amandine Spiandore, Erik H. Rikkerink, Matthew D. Templeton, Cécile Segonzac, Kee Hoon Sohn

**Affiliations:** 1 Bioprotection Research Centre, Institute of Agriculture and Environment, Massey University, Palmerston North, 4474 New Zealand; 2 New Zealand Institute for Plant & Food Research Limited (PFR), Private Bag, 92169 Auckland, New Zealand; 3 School of Biological Sciences, University of Auckland, Private Bag, 92019 Auckland, New Zealand; 4 Department of Plant Science, Plant Genomics and Breeding Institute, College of Agriculture and Life Sciences, Seoul National University, Seoul, 08826 Republic of Korea; 5 Department of Life Sciences, Pohang University of Science and Technology, Pohang, 37673 Republic of Korea; 6 School of Interdisciplinary Bioscience and Bioengineering, Pohang University of Science and Technology, Pohang, 37673 Republic of Korea

## Abstract

Type-III secreted effectors (T3Es) play critical roles during bacterial pathogenesis in plants. Plant recognition of certain T3Es can trigger defence, often accompanied by macroscopic cell death, termed the hypersensitive response (HR). Economically important species of kiwifruit are susceptible to *Pseudomonas syringae* pv. *actinidiae* (*Psa*), the causal agent of kiwifruit bacterial canker. Although *Psa* is non-pathogenic in *Arabidopsis thaliana*, we observed that a T3E, HopZ5 that is unique to a global outbreak clade of *Psa*, triggers HR and defence in Arabidopsis accession Ct-1. Ws-2 and Col-0 accessions are unable to produce an HR in response to *Pseudomonas*-delivered HopZ5. While Ws-2 is susceptible to virulent bacterial strain *Pseudomonas syringae* pv. *tomato* DC3000 carrying HopZ5, Col-0 is resistant despite the lack of an HR. We show that HopZ5, like other members of the YopJ superfamily of acetyltransferases that it belongs to, autoacetylates lysine residues. Through comparisons to other family members, we identified an acetyltransferase catalytic activity and demonstrate its requirement for triggering defence in Arabidopsis and *Nicotiana* species. Collectively, data herein indicate that HopZ5 is a plasma membrane-localized acetyltransferase with autoacetylation activity required for avirulence.

## Introduction

Plants resist microbial invasion through innate defence systems including a first layer of inducible immunity that recognizes conserved pathogen-associated molecular patterns (PAMPs). Plant cell surface-bound pattern recognition receptors (PRRs) bind to corresponding PAMPs and initiate PAMP-triggered immunity (PTI)^1^. Successful bacterial pathogens can suppress PTI through the use of a type III secretion system (T3SS) to inject proteinaceous virulence factors, termed effectors (T3E), directly into plant cells^2^. In order to suppress PTI, bacterial T3Es possess a wide variety of enzymatic functions that target multiple components of the PTI signalling pathway^3^. On the other hand, plants carry intracellular nucleotide-binding and leucine-rich repeat resistance proteins (NLR) that can sense the presence of one or more effectors to activate a second layer of defence, effector-triggered immunity (ETI)^4^, that restricts pathogen growth. A recognized effector that triggers ETI is thus termed an avirulence protein (Avr). ETI is often accompanied by rapid transcriptional reprogramming of defence genes, production of antimicrobial compounds and the hypersensitive response (HR), a localized programmed cell death at the site of infection that serves to restrict pathogen spread^4^.

NLR proteins consist of a central nucleotide-binding domain, a carboxy-terminal leucine-rich repeat domain and a variable amino-terminal domain, often a toll-interleukin receptor (TIR)-type or coiled-coil (CC)-type, hereafter referred to as TNLs and CNLs, respectively^5^. NLRs characteristically require common downstream signalling components, including the lipase-like protein ENHANCED DISEASE SUSCEPTIBILITY 1 (EDS1) or the integrin-like protein NON-RACE SPECIFIC DISEASE RESISTANCE 1 (NDR1) involved in cell wall-plasma membrane adhesion for some TNL and CNL classes, respectively^6–8^. In addition to these two branches of NLR signalling, a shared component involves the *SUPPRESSOR OF THE G2 ALLELE OF SKP1* (*SGT1*) gene. SGT1 is a highly conserved protein in plants that participates in a chaperone complex with HEAT SHOCK PROTEIN 90 (HSP90) and REQUIRED FOR MLA12 RESISTANCE 1 (RAR1) to maintain NLR homeostasis and was shown to be integral to defence mediated by various NLRs^9, 10^. NLRs have also shown a requirement for the phytohormone salicylic acid (SA) for the proper induction of systemic acquired resistance (SAR) following NLR activation, a system requiring the SA biosynthesis gene *SID2* ^11, 12^.

*Pseudomonas syringae* pv. *actinidiae* (*Psa*) causes a bacterial canker disease in kiwifruit and has been a global threat to the kiwifruit industry since the 1980s and consists of several distinct clades known as biovars. The source of a recent global outbreak of bacterial canker disease in kiwifruit orchards in major exporters like Italy (2008), Chile (2010) and New Zealand (2010) has been identified as biovar 3 strains of *Psa* which includes the NZV-13 (ICMP 18884, CP011972–3) strain from New Zealand^13^. The *Psa* NZV-13 genome was recently sequenced in order to understand its origin and virulence mechanisms^14, 15^. Similar to other *P*. *syringae* strains, *Psa* NZV-13 carries the genes encoding the T3SS and a repertoire of 34 predicted T3Es. Putative biochemical activities of *Psa* NZV-13 T3Es include ribosyltransferase, glutamidase, phospholyase, cysteine protease, and acetyltransferase. Interestingly, HopZ5 (AKT29515.1), a putative acetyltransferase, and HopH1 (AKT29516.1) are uniquely present among the global outbreak strains of biovar 3 including *Psa* NZV-13^14^.

The YopJ (Yersinia outer protein J) effector protein from the human gastroenteritis pathogen *Yersinia pseudotuberculosis* was shown to have an acetyltransferase activity. YopJ acetylates multiple MITOGEN ACTIVATED PROTEIN KINASE KINASE (MAPKK) proteins, key components of host defense, that results in inhibition of their phosphorylation and suppression of NFκB mammalian defense signalling pathways^16, 17^. Interestingly, plant pathogenic bacteria carry a number of YopJ-class T3Es including *Ralstonia* PopP2 and the *Pseudomonas* HopZ family of effectors^18, 19^. HopZ1a from *P*. *syringae* pv. *syringae* A2 autoacetylates a specific threonine residue (T346) with the involvement of two serine residues (S349 and S351) following *in planta* activation of HopZ1a by the plant cofactor IP6^20, 21^. In addition, HopZ1a acetylates jasmonate ZIM-domain (JAZ) repressors of jasmonic acid (JA) signalling leading to their proteosomal degradation, thereby activating the JA pathway and antagonizing salicylic acid-mediated disease resistance^22^. Additionally, HopZ1a targets tubulin to block defence at the cell wall while simultaneously interfering with the host’s secretory pathway that has been linked to systemic immunity^23–25^. However, in the presence of a functional CNL gene, *ZAR1*, HopZ1a activates ETI in *Arabidopsis thaliana* (hereafter referred to as Arabidopsis)^26^. HopZ1a-triggered immunity requires Arabidopsis pseudokinase ZED1 that is monitored by ZAR1^27^. The downstream genetic components required for HopZ1a-triggered immunity remains elusive^26, 27^. The *Ralstonia solanacearum* GMI1000 effector PopP2 is a nucleus-localized acetyltransferase, that autoacetylates a lysine residue (K383) required for its *trans*-acetylation activity^18^ and targets WRKY transcription factors involved in defence activation^28, 29^. The recognition of PopP2 in Arabidopsis is conferred by the TNL protein pair RRS1 and RPS4. PopP2 acetylates the WRKY DNA-binding domain of RRS1 resulting in destabilized RRS1 binding to its target DNA^28–30^. It is hypothesized that PopP2 targets other WRKY transcription factors to enhance bacterial virulence yet a detailed mechanism is unknown. Based on these findings, despite their conserved enzymatic activity, different members of the YopJ acetyltransferase family may have been specialized for targeting distinct plant defence components.

In this study we sought to examine the genetic basis of the *Psa* NZV-13-triggered HR in Arabidopsis. By using T3SS delivery of *Psa* effectors from *P*. *fluorescens* Pf0-1 (T3S), a non-pathogenic strain engineered to carry a functional T3SS^31^, we found that HopZ5 triggers ETI in Arabidopsis. By analogy with other HopZ members, HopZ5 encodes a putative acetyltransferase and we show that this enzymatic activity is required for its avirulence activity. In addition, HopZ5-induced cell death in *Nicotiana benthamiana* requires *SGT1* but not *EDS1* or *NDR1*. Furthermore, we discovered that HopZ5 triggers ETI that is not associated with HR development in an Arabidopsis accession-specific manner.

## Results

### HopZ5_*Psa*NZV-13_ triggers accession-specific immunity in Arabidopsis

**Figure 1:**
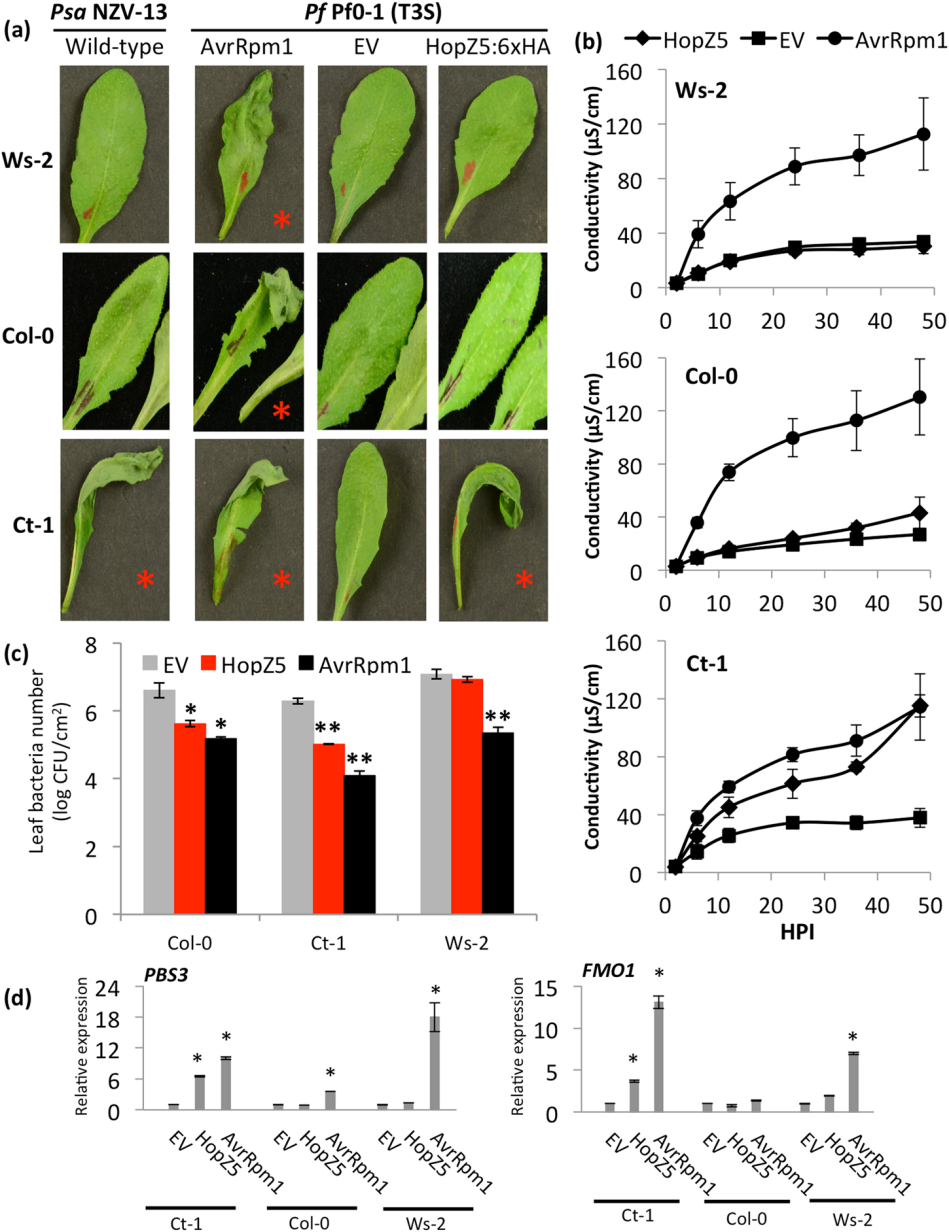
*Pseudomonas syringae* pv. *actinidiae* type III effector HopZ5 triggers accession-specific immunity in Arabidopsis. (**a**) Arabidopsis accession-dependent development of a hypersensitive response to *P*. *syringae* pv. *actinidiae* and its type-III secreted effector HopZ5. Leaves of Arabidopsis plants were infiltrated with *P*. *syringae pv*. *actinidiae* NZV-13 *or P*. *fluorescens* Pf0-1 (T3S) carrying an empty vector pBBR1MCS-5 (EV), *hopZ5* with a C-terminal 6xHA tag under the control of the *avrRps4* promoter in pBBR1MCS-5 or untagged *avrRpm1* under its native promoter in pVSP61. Bacterial suspensions (1 × 10^8^ CFU/mL) were blunt-syringe infiltrated into the leaves and photographs were taken 20 hours after infiltration (1 dpi). Development of the hypersensitive response is indicated by a red asterisk. (**b**) Electrolyte leakage from Arabidopsis leaf discs after infiltration with *P*. *fluorescens* Pf0-1 (T3S) carrying the indicated constructs, as in (**a**). Bacteria (1 × 10^8^ CFU/mL) were blunt syringe-infiltrated into the leaves. The error bars indicate the standard error from four technical replicates. The experiment was conducted three times with similar results. (**c**) *P*. *syringae* pv. *tomato* DC3000 (*Pto* DC3000) growth in Arabidopsis. *Pto* DC3000 carrying EV, *hopZ5*-*HA* or *avrRpm1* was blunt syringe-infiltrated at 5 × 10^5^ CFU/mL into Arabidopsis leaves, and bacterial growth was determined 4 days post-infection (4 dpi). Error bars represent standard error from six technical replicates. Asterisks indicate results of Student’s *t*-test between selected sample and EV for that accession; *(*P* < 0.05), **(*P* < 0.01). The experiment was conducted six times with similar results. (**d**) Defence gene expression in Arabidopsis in response to EV, HopZ5-HA or AvrRpm1. *P*. *fluorescens* Pf0-1 (T3S) (1 × 10^8^ CFU/mL) carrying EV, *hopZ5*-*HA* or *avrRpm1* was blunt syringe-infiltrated into leaves, samples taken at 8 hours post infiltration and defence gene expression determined from extracted RNA by quantitative polymerase chain reaction. Expression for each defence gene is relative to internal *EF1α* expression and defence gene expression for EV samples indicated. Error bars indicate standard error from three technical replicates. Asterisks indicate results of Student’s t-test between selected sample and EV for that accession; *(*P* < 0.01). The experiment was conducted three times with similar results.

Several *P*. *syringae* type-III secreted effectors (T3E) were shown to trigger *NLR*-dependent immunity in Arabidopsis^26, 32–34^. To examine if *P*. *syringae* pv. *actinidiae* (*Psa*) NZV-13 can also trigger immunity in Arabidopsis, we screened several accessions for *Psa* NZV-13-triggered hypersensitive response (HR) which is typically associated with effector-triggered immunity (ETI)^35^. *Psa* NZV-13 triggered a strong HR in the Catania-1 (Ct-1) accession but not in Col-0 and Wassilewskija-2 (Ws-2) (Fig. 1a). In order to identify the *Psa* NZV-13 effector that triggers HR in Ct-1, we cloned all 34 predicted T3Es (based on tBLASTx annotation followed by manual confirmation as done previously^14^) from *Psa* NZV-13 into a broad host-range vector (pBBR 1MCS-5) with expression driven by the previously characterized *avrRps4* promoter^33, 36^. Individual T3E delivery by *Pseudomonas fluorescens* (*Pf*) Pf0-1(T3S) (hereafter, Pf0-1(T3S))^31^ identified HopZ5 as a cause of the HR in Ct-1 (Fig. 1a). Col-0 and Ws-2 did not develop HR in response to Pf0-1(T3S)-delivered HopZ5 or empty vector (EV). However, as expected, Pf0-1(T3S)-delivered AvrRpm1 (from *P*. *syringae* pv. *maculicola* M6) triggered HR in all three Arabidopsis accessions tested, indicating that our experimental conditions were optimal. To quantify HopZ5-triggered HR, we measured the leakage of ions caused during HR development in the infected leaves. As expected, Pf0-1(T3S)-delivered AvrRpm1 induced significant increase in electrolyte leakage levels in all three Arabidopsis accessions tested whereas EV did not cause a notable change. Consistent with HR data, Pf0-1(T3S)-delivered HopZ5-triggered significantly increased electrolyte leakage level as compared to EV in Ct-1 but not in Ws-2 nor Col-0 (Fig. 1b).

Next, we assessed the effect of HopZ5 on *in planta* growth of virulent *P*. *syringae* pv. *tomato* (*Pto*) DC3000. *Pto* DC3000 strains carrying *hopZ5* or *avrRpm1* grew significantly less (a reduction of one log) than EV in Ct-1 (Fig. 1c). Interestingly, although neither Col-0 nor Ws-2 accessions show HR in response to Pf0-1(T3S)-delivered HopZ5, we consistently observed a small but significant growth restriction in Col-0 (a reduction of almost one log) but not in Ws-2. To further examine the HR-deficient immunity in Col-0, we assessed HopZ5-triggered defence gene expression by Pf0-1(T3S)-delivery. Surprisingly, Pf0-1(T3S)-delivered HopZ5 upregulated transcript accumulation of two previously characterized defence genes *PBS3* and *FMO1* in Ct-1 but not in Col-0 and Ws-2 (Fig. 1d). Since HopZ5 restricts *in planta Pto* DC3000 growth in both Ct-1 and Col-0, we tested whether *Pto* DC3000 delivery of HopZ5 induces defence marker gene expression. As expected, Col-0 showed upregulated *PBS3* and *FMO1* transcript accumulation in response to *Pto* DC3000-delivered HopZ5 and AvrRpm1 compared to EV (Supplementary Fig. S1). These data suggest that HopZ5 triggers defence response in Ct-1 and Col-0.

### HopZ5 triggers an *NbSGT1-*dependent hypersensitive response-like cell death in *Nicotiana benthamiana*

**Figure 2:**
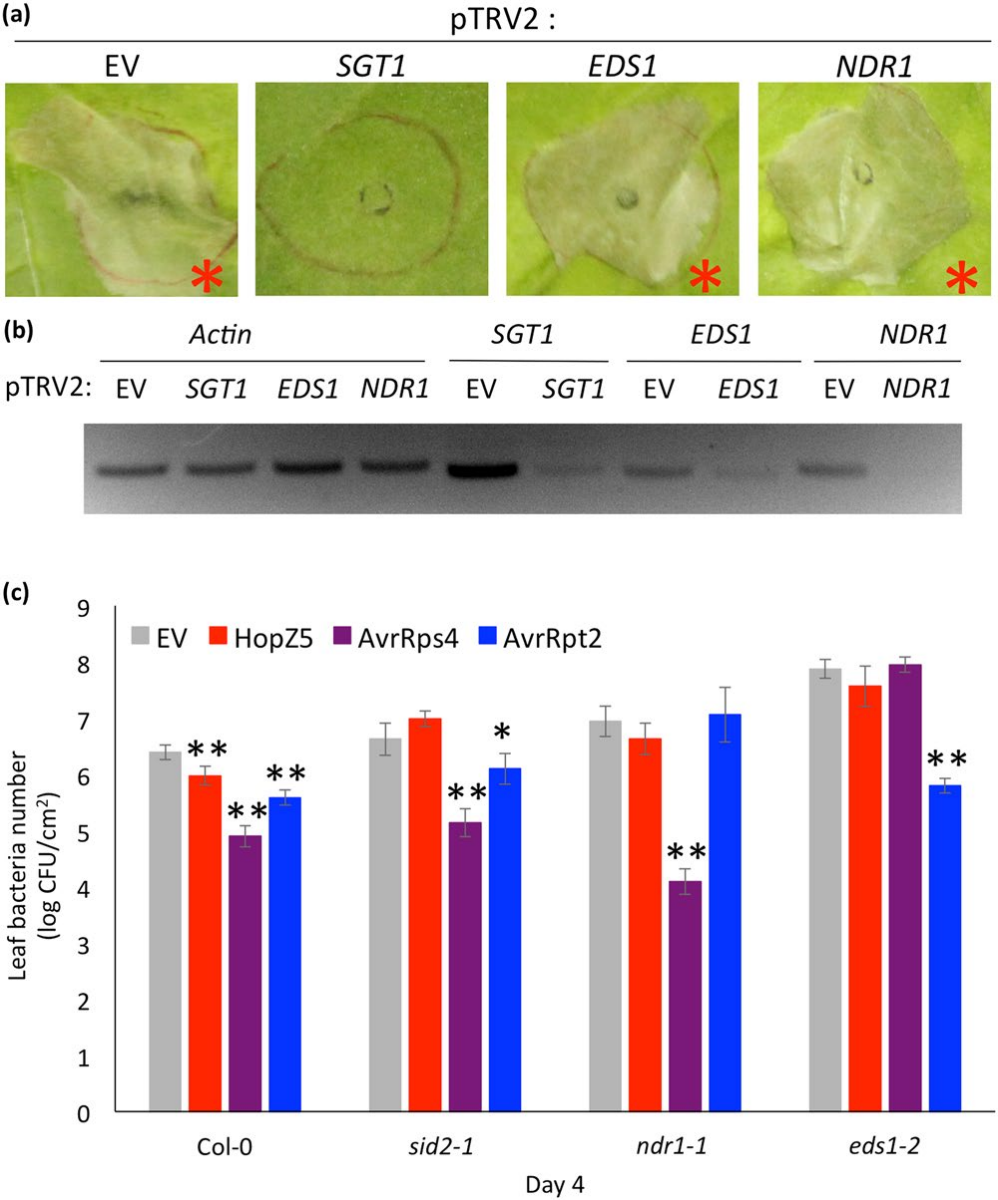
HopZ5 recognition occurs in *Nicotiana benthamiana* and requires *SGT1* but not *NDR1* or *EDS1*. (**a**) *SGT1*-silenced *N. benthamiana* loses HopZ5-triggered hypersensitive response. Two-week-old *N*. *benthamiana* seedlings were infiltrated with *Agrobacterium tumefaciens* AGL1 carrying pTRV2 targeting *SGT1*, *EDS1* or *NDR1* for silencing, or the empty pTRV2 vector alone. 4–5 weeks post VIGS treatment, YFP-tagged variants of HopZ5 was transiently expressed and photographs taken at 3 days post infiltration. Red asterisks indicate development of cell death symptoms. The experiment was conducted three times with similar results. (**b**) Silencing of target genes was confirmed by RNA extraction from multiple leaves followed by semi-quantitative polymerase chain reaction. Actin was used as control for RNA. Labels above indicate the gene tested for expression by semi-qPCR while labels below indicate the silencing construct used. (**c**) *Pto* DC3000 carrying EV, *hopZ5*-*HA*, *avRps4* or *avrRpt2* was blunt syringe-infiltrated at 5 × 10^5^ CFU/mL into leaves of Arabidopsis wildtype Col-0, *sid2-1*, *ndr1-1*, or *eds1-2* mutant lines. Bacterial growth was determined 4 days post-infection (4 dpi). Error bars represent standard error from six technical replicates. Asterisks indicate results of Student’s *t*-test between selected sample and EV for that accession; *(*P* < 0.05), **(*P* < 0.01). The experiment was conducted three times with similar results.

Agrobacterium-mediated transient expression (hereafter, agroinfiltration) of pathogen effectors in *Nicotiana* spp. often induces HR-like cell death (HCD)^37, 38^. In order to test if HopZ5 triggers HCD in *Nicotiana* spp., we transiently expressed C-terminally YFP-tagged HopZ5 under the control of constitutive cauliflower mosaic virus 35 S promoter (35 S promoter) in *Nicotiana benthamiana* via agroinfiltration. Interestingly, HopZ5 triggered a robust HCD within 2 dpi (Fig. 2a). We used a well-established virus-induced gene silencing (VIGS) system to examine the genetic components required for HopZ5-triggered HCD in *N*. *benthamiana*. We generated VIGS constructs for *NbEDS1*, *NbNDR1* and *NbSGT1* as they have been previously identified as critical components in rendering HCD^39, 40^. While silencing enabled significant reduction in transcriptional expression of *NbEDS1*, *NbNDR1* and *NbSGT1* compared to the empty vector silencing construct, only *NbSGT1*-silenced plants lost the ability to mount HCD in response to HopZ5 expression (Fig. 2a,b). This result suggests that the HopZ5-triggered activation of defence signalling, at least in *N*. *benthamiana*, requires *SGT1* but not *EDS1* or *NDR1*.

To test if this lack of requirement for *NDR1* and *EDS1* is reproduced in Arabidopsis, we tested if Col-0 mutants, *eds1-2*, *ndr1-1* and *sid2-1*, were able to restrict growth of *Pto* DC3000 delivering HopZ5 or two different avirulence effectors, AvrRps4 or AvrRpt2^6, 11, 12^. Surprisingly, in contrast to wild-type Col-0, all three mutants (*sid2-1*, *ndr1-1* and *eds1-2*) were affected in their ability to restrict growth of *Pto* DC3000 delivering HopZ5 (Fig. 2c). As expected, resistance to *Pto* DC3000 expressing AvrRpt2 or AvrRps4 was abolished in *ndr1-1* or eds1-2, respectively^6^.

### Plasma membrane localization and autoacetylation are required for HopZ5 avirulence activity

**Figure 3:**
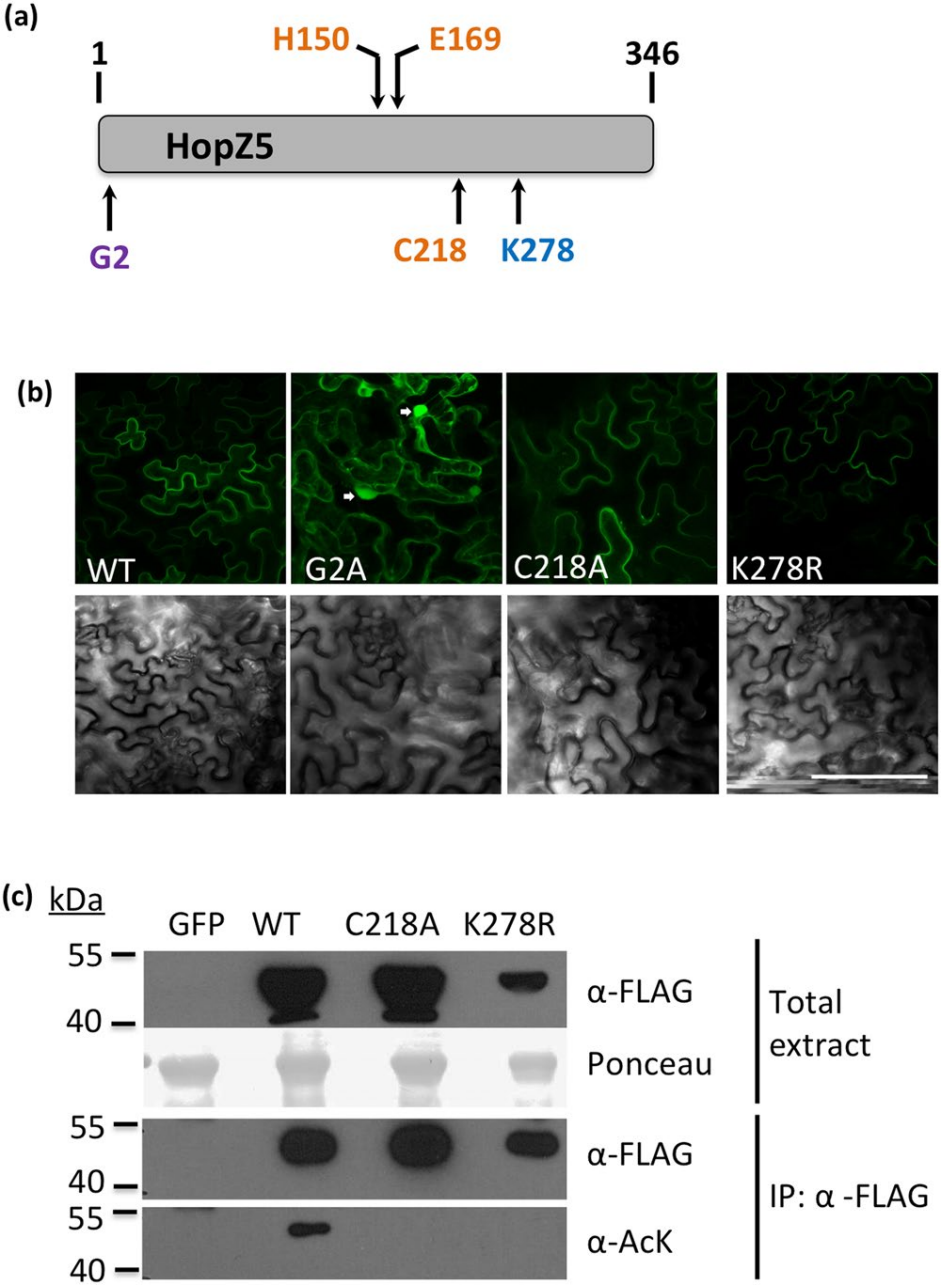
Plasma membrane localization and autoacetylation are affected in HopZ5 variants. (**a**) Key residues for HopZ5 function predicted through conservation between YopJ family members. Line model for HopZ5 peptide with putative myristoylation residue (glycine, G2), acetyltransferase catalytic core residues (histidine, H150; glutamate, E169; cysteine, C218) and autoacetylation-required site (lysine, K278) is shown. Mutants generated in this study include a glycine to alanine mutation (G2A), cysteine to alanine (C218A) and a lysine to arginine (K278R). (**b**) *In planta* subcellular localization of HopZ5 variants was determined in *SGT1*-silenced *N*. *benthamiana*. Leaves were infiltrated with *Agrobacterium tumefaciens* AGL1 carrying YFP-tagged *hopZ5* (WT), *hopZ5* (G2A), *hopZ5* (C218A) or *hopZ5* (K278R) under a constitutive CaMV 35 S promoter. Leaf discs were taken at 2 days post infiltration and visualized under confocal laser-scanning microscopy for localization of the YFP tag. Top and bottom panels are confocal images and corresponding bright-field images, respectively. White arrows in G2A sample indicate plant cell nuclei. Scale bar represents 50 μm. (**c**) Detection of acetylated wild-type HopZ5 (WT), but not HopZ5-C218A and HopZ5-K278R from transient expression in *N*. *benthamiana SGT1*-silenced plants. Protein was extracted from leaf samples 2 days post infiltration. Protein presence in total extract was tested by α-FLAG antibody and acetylation status tested by immunoblot with an α-AcK antibody after α- FLAG immunoprecipitation. Ponceau Red staining of total protein extract shows sample loading.

*In silico* analysis of HopZ5 suggests that it belongs to the YopJ superfamily of acetyltransferases and cysteine proteases (Supplementary Fig. S2). Several members of this family including *Pseudomonas syringae* HopZ1, *Xanthomonas campestris* AvrBsT and *R*. *solanacearum* PopP2 have been characterized previously^18, 26, 41^. HopZ5, similar to other YopJ family members, possesses several conserved key residues including a catalytic triad consisting of histidine (H150), glutamate (E169) and cysteine (C218) and a lysine residue putatively required for auto-/trans-acetylation (K278) (Fig. 3a; Supplementary Fig. S3). Additionally, HopZ5 possesses a glycine (G2) that is conserved in most members of the HopZ family of T3Es and required for myristoylation-dependent plasma membrane localization^42^. The phylogenetic relationships and predicted conserved residues suggest that HopZ5 may be a plasma membrane-localized acetyltransferase.

Similar to the work carried out for PopP2^18^ and HopZ1a^42, 43^ for which critical amino acid residues were mutagenized to analyse biochemical functions, we utilized site-directed mutagenesis to generate HopZ5 variants carrying a C218A (cysteine to alanine) or K278R (lysine to arginine) mutation that would eliminate the predicted acetyltransferase activity or autoacetylation, respectively. We also generated a G2A (glycine to alanine) HopZ5 variant to alter the predicted subcellular localization. To confirm the biochemical requirement for these key residues, we then used *NbSGT1-*silenced *N*. *benthamiana* since HopZ5-triggered HCD was reduced in these plants, promoting HopZ5 protein accumulation and detection. Agroinfiltration of C-terminally YFP-tagged HopZ5 (HopZ5-YFP) variants allowed detection of subcellular localization of HopZ5 by confocal microscopy. We found that while wild-type HopZ5 and both C218A and K278R variants localized to the cell periphery, the G2A variant localized in the cytoplasm and nuclei of *Agrobacteria*-infected cells (Fig. 3b, Supplementary Fig. S4). The presence of YFP signal in the cytoplasmic strands in G2A infiltrated plants as well as in nuclei (white arrows in Fig. 3b, and red arrows colocalized with DAPI stain in Supplementary Fig. S4) suggests that wild-type HopZ5 normally localizes at the plant plasma membrane. Several *R*. *solanacearum* effector PopP2 was shown to autoacetylate lysine residues^18^. The C218A and K278R variants were thus further examined for their ability to acetylate internal lysine residues, as an indicator of autoacetylation ability. Transiently expressed FLAG-tagged HopZ5 variants were immunoprecipitated and probed with an antibody that detects acetylated lysine residues. We found that wild-type HopZ5 but not the C218A variant was lysine-acetylated, suggesting that C218A has lost its acetyltransferase activity and that it is autoacetylated (Fig. 3c). In addition, although catalytic residues (H150, E169 and C218) are present, lysine-acetylation of the K278R variant was not detectable, indicating either that it may be the sole acetylated lysine residue and is autoacetylated in HopZ5, or that it is required for autoacetylation in general.

**Figure 4:**
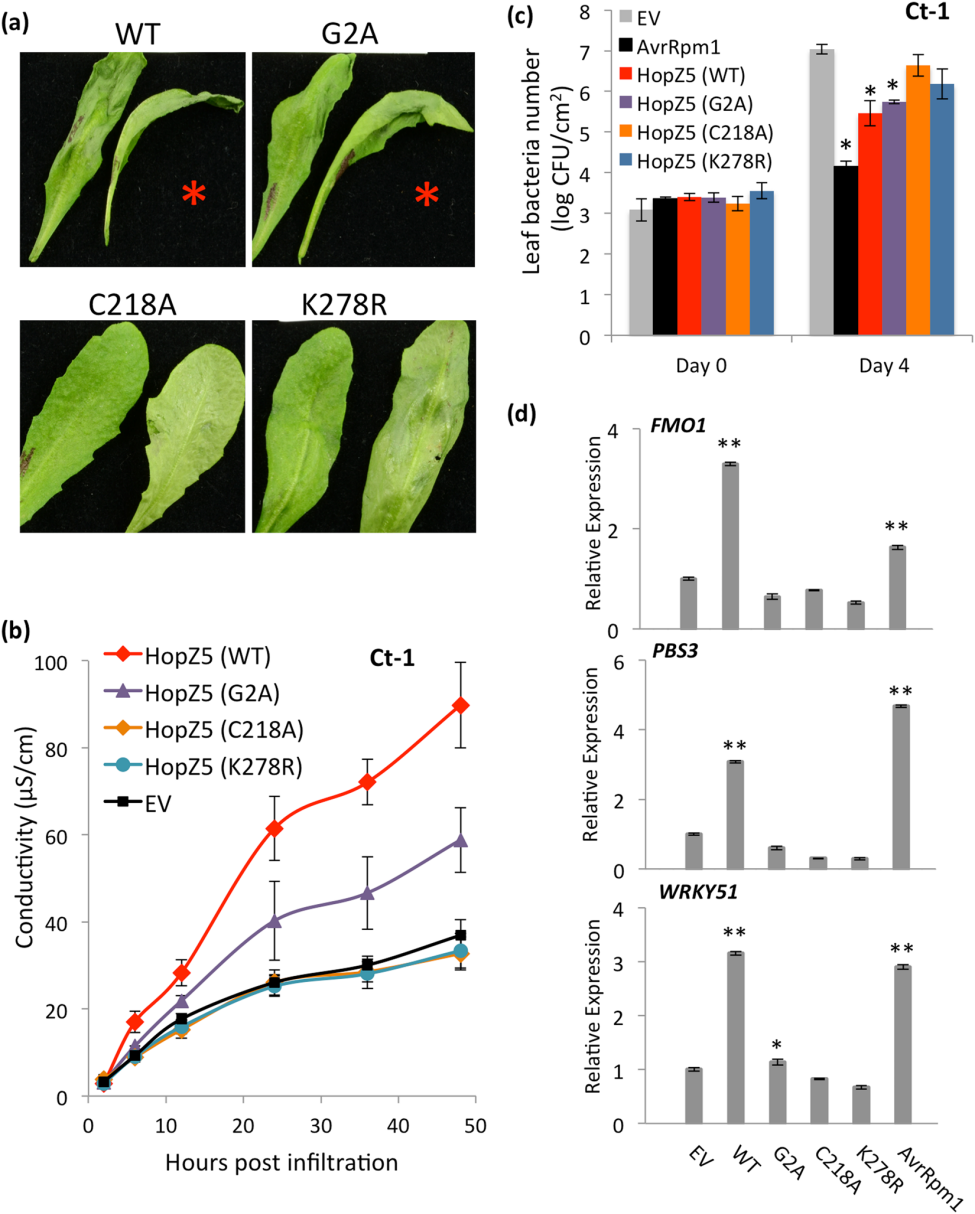
Putative acetyltransferase activity and myristoylation of HopZ5 are required for avirulence. (**a**) Development of the hypersensitive response in the Arabidopsis accession Ct-1 against HopZ5 variants. *P*. *fluorescens* Pf0-1 (T3S) (1 × 10^8^ CFU/mL) carrying *hopZ5*-*HA* (WT), *hopZ5*(G2A)-*HA*, *hopZ5*(C218A)-*HA* or *hopZ5*(K278R)-*HA* was blunt syringe-infiltrated into leaves and photographs taken 20 hours after infiltration. The red asterisk indicates development of the hypersensitive response. (**b**) Electrolyte leakage from Arabidopsis accession Ct-1 leaf discs after infiltration with *P*. *fluorescens* Pf0-1 (T3S) expressing HopZ5 variants, as in (**a**). Bacteria (1 × 10^8^ CFU/mL) were blunt syringe-infiltrated into the leaves. The error bars indicate the standard error from four technical replicates. The experiment was conducted five times with similar results. (**c**) *Pto* DC3000 growth in Arabidopsis accession Ct-1. *Pto* DC3000 carrying *hopZ5* variants, as in (**a**), was blunt syringe-infiltrated at 5 × 10^5^ CFU/mL into Arabidopsis leaves, and bacterial growth was determined 4 days post-infection (4 dpi). Error bars represent standard error from six technical replicates. Asterisks indicate results of Student’s *t*-test between selected sample and EV for that accession; *(*P* < 0.05), **(*P* < 0.01). The experiment was conducted nine times with similar results. (**d**) Defence gene expression in Arabidopsis Ct-1 in response to HopZ5 variants, as in (**a**). *P*. *fluorescens* Pf0-1 (T3S) (1 × 10^8^ CFU/mL) carrying the indicated construct was blunt syringe-infiltrated into leaves and defence gene expression determined from extracted RNA by quantitative polymerase chain reaction. Expression for each defence gene is relative to internal *EF1α* expression and defence gene expression for EV samples indicated. Error bars indicate standard error from three technical replicates. Asterisks indicate results of Student’s t-test between selected sample and EV for that accession; *(*P* < 0.05), **(*P* < 0.01). The experiment was conducted five times with similar results.

To determine if plasma membrane localization and autoacetylation of HopZ5 are required for avirulence, we examined the defence responses triggered by HopZ5 variants in the resistant Arabidopsis accession Ct-1. Pf0-1(T3S)-delivered HopZ5 C218A or K278R variants did not trigger HR in Ct-1 leaves (Fig. 4a). Interestingly, the G2A variant only showed a partial loss of HR as observed through a qualitative assessment of leaf appearance (Fig. 4a). Electrolyte leakage quantitation showed a significant but not complete attenuation of ion leakage for the G2A variant while both C218A and K278R variants induced a much reduced level, similar to EV (Fig. 4b). Next, these HopZ5 variants were assessed for their ability to restrict growth of a virulent bacterial strain *Pto* DC3000 in Ct-1. Although the G2A variant induced significantly reduced ion leakage as compared to wild-type HopZ5 (Fig. 4b), *in planta* growth of *Pto* DC3000 carrying G2A variant was comparable to *Pto* DC3000 carrying wild-type *hopZ5* (Fig. 4c). In addition, both C218A and K278A variants did not significantly restrict *Pto* DC3000 growth compared to EV. To further elucidate the early defence responses triggered by HopZ5 variants, the expression of several defence marker genes was determined. Pf0-1(T3S)-delivered wild-type HopZ5 but not G2A, C218A or K278A variants induced significant accumulation of defence marker gene expression as compared to EV (Fig. 4d). All three variants of HopZ5 were unaffected in protein expression and stability in *Pto* DC3000, indicating that the differences seen between wild-type HopZ5 and the variants in their ability to trigger immunity was not a result of inconsistent protein expression due to the mutations (Supplementary Fig. S5). Taken together, these data suggest that all three predicted functional residues are at least partially required for a full induction of ETI.

**Figure 5:**
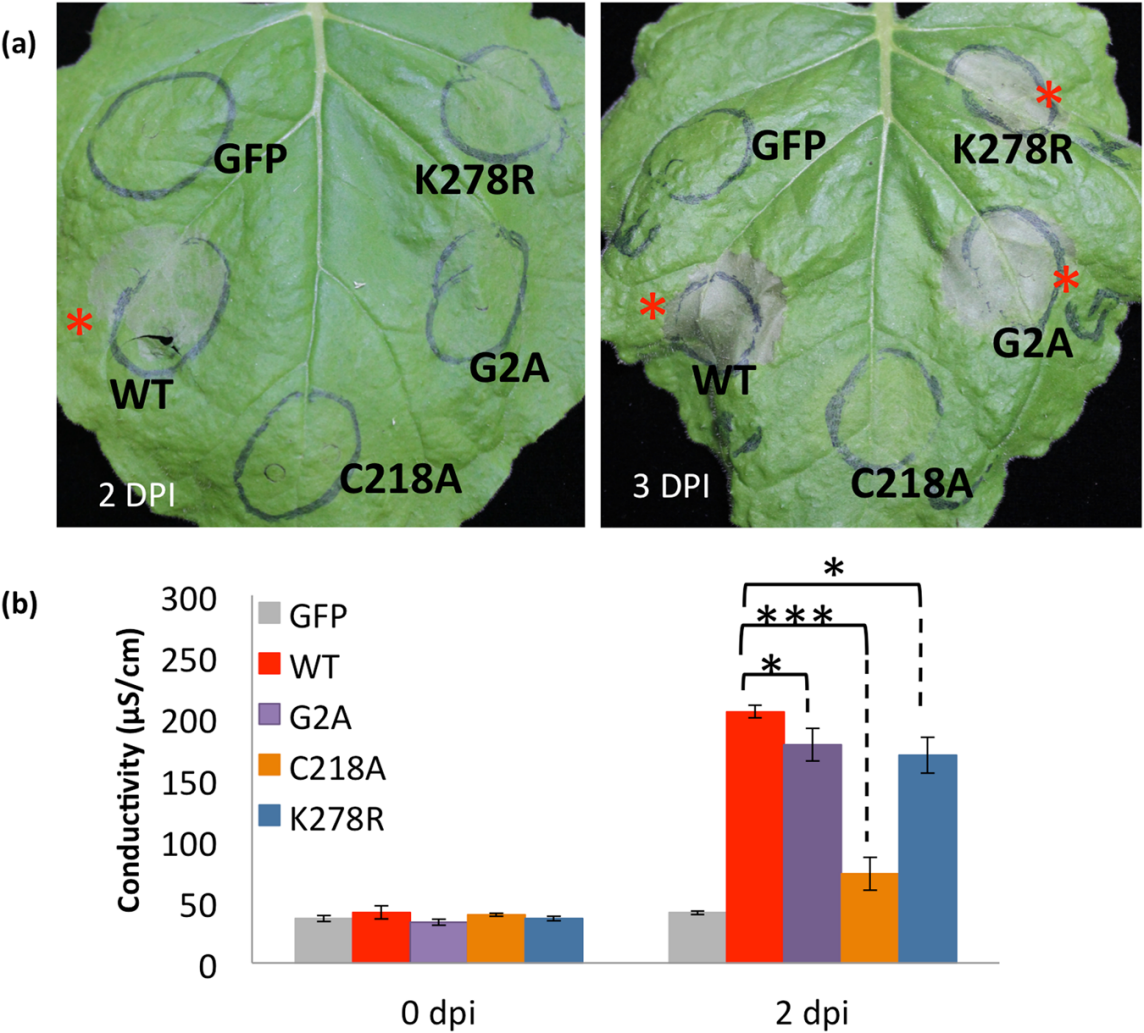
HopZ5 recognition in *Nicotiana benthamiana* requires acetyltransferase activity. (**a**) HopZ5 (WT) or mutated myristoylation (G2A), catalytic core (C218A) and autoacetylation-required (K278R) variants tagged with 3xFLAG were transiently expressed under the CaMV 35 S promoter in *N*. *benthamiana* Photographs were taken at 2 or 3 days post infiltration (dpi). Red asterisks indicate development of hypersensitive response-like cell death symptoms. This experiment was conducted six times with identical results. (**b**) Electrolyte leakage from *N*. *benthamiana* leaf discs triggered by *Agrobacterium*-mediated transient expression of HopZ5 or variants, as in (**a**), was determined at 0 and 2 days post infiltration (dpi). Infiltrations were carried out by blunt syringe at OD_600_ = 0.4 for all samples. Error bars represent standard error from four technical replicates. Asterisks indicate results of Student’s *t*-test between selected samples; *(*P* < 0.05), ***(*P* < 0.001). This experiment was conducted three times with identical results.

In order to test if HopZ5-triggered defence responses in other plant species require similar properties of HopZ5, HopZ5-YFP variants were transiently expressed in *N*. *benthamiana* and *N*. *tabacum*. Agroinfiltration of wild-type HopZ5 but not GFP control induced rapid development of HCD in both *N*. *benthamiana* (Fig. 5a) and *N*. *tabacum* (Supplementary Fig. S6). Consistent with *Pseudomonas*-delivered HopZ5 results in Arabidopsis, the C218A variant did not induce any visible cell death. Interestingly, G2A and K278R variants induced delayed cell death development, indicating that avirulence activity of these variants was significantly reduced. To quantify the effect of the mutations on HopZ5-triggered HCD, we carried out an electrolyte leakage assay after agroinfiltration of G2A, C218A and K278R variants in *N*. *benthamiana* (Fig. 5b, Supplementary Fig. S7). Electrolyte leakage measurement at 2 days post-inoculation (dpi), when HCD symptoms started to appear prior to leaf collapse, demonstrated a statistically significant reduction for both the G2A and K278R variants and a complete loss of HCD-associated leakage for the C218A variant. These results demonstrate that the partial loss of avirulence function for HopZ5 G2A and K278A variants applies in more than one plant host (summarized in Supplementary Table 1).

### HopZ5 triggers defence that is not associated with HR in Col-0

**Figure 6:**
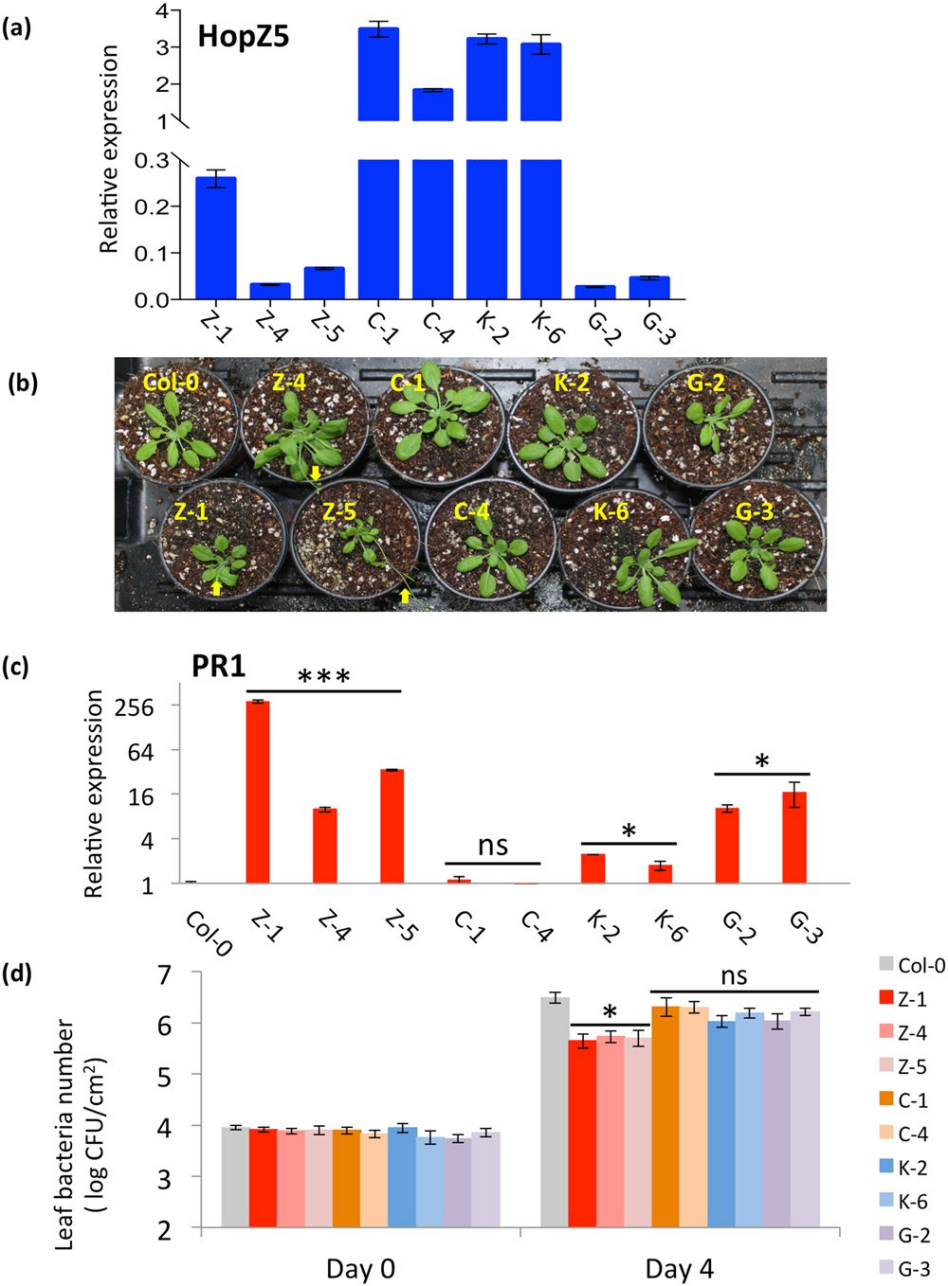
HopZ5 variants are compromised in their ability to trigger defence when overexpressed in *Arabidopsis thaliana* Col-0. (**a**) *hopZ5*-transgene expression was determined in *hopZ5* stable expression lines in the Col-0 background. 15–20 T2 seedlings from two or three independent lines per *hopZ5* variant (*hopZ5*-wild-type: Z-1, Z-4, Z-5; *C218A*: C-1, C-4; *K278R*: K-2, K-6; *G2A*: G-2, G-3) were harvested after selection for three weeks on solid media containing kanamycin. RNA was extracted and cDNA synthesized and used for quantitative polymerase chain reaction (qPCR) for the *hopZ5-YFP* transgene. Expression is relative to internal *EF1α* expression only. Error bars indicate standard error from three technical replicates. Asterisks indicate results of Student’s t-test between selected sample and Col-0 wild-type also grown on plates but without kanamycin; ***(*P* < 0.0001). This experiment was conducted three times with similar results. (**b**) Stable transgenic lines expressing wild-type HopZ5 show altered morphology. 2-week-old T2 plants as in (**a**) were transferred to soil after selection on kanamycin. Photographs are of representative 5-week-old plants that showed a differential phenotype only. Typically between 1-in-3 (Z-1) to 1-in-4 plants (Z-4 and Z-5) showed a stunted/early flowering phenotype, putatively representative of Mendelian segregation and individuals homozygous for the transgene. Yellow arrows indicate the inflorescence in the early-flowering plants. (**c**) *PR1* marker gene expression was determined in *hopZ5* stable expression lines as in (**a**). Expression is relative to internal *EF1α* expression and *PR1* expression for wildtype. Error bars indicate standard error from three technical replicates. Asterisks indicate results of Student’s t-test between selected sample and Col-0 wild-type also grown on plates but without kanamycin; ***(*P* < 0.0001). This experiment was conducted three times with similar results. (**d**) *Pto* DC3000 growth in *hopZ5* stable expression lines in Col-0. *Pto* DC3000 was blunt syringe-infiltrated at 5 × 10^5^ CFU/mL into leaves of each stable expression line from (**a**) and bacterial growth was determined 4 days post-infection (4 dpi). Error bars represent standard error from six technical replicates. Asterisks indicate results of Student’s *t*-test between selected sample and Col-0 wild-type; ns (*P* > 0.05), *(*P* < 0.05), **(*P* < 0.01). The experiment was conducted three times with similar results.

To clarify the HopZ5-triggered bacterial immunity without HR in Col-0 plants, we generated stable transgenic Col-0 lines expressing HopZ5-YFP variants under the control of the 35 S promoter. Using multiple independent lines for wild-type HopZ5 (Z-1, Z-4, Z-5), C218A (C-1, C-4), K278R (K-2, K-6) and G2A (G-2, G-3), we first tested for expression of the transgene in T2 plants. Notably, we were only able to identify low-expression lines for wild-type HopZ5 (Z) and G2A (G) variants while C218A (C) and K278R (K) variant lines had significantly higher transgene expression levels (Fig. 6a). Additionally, T2 plants for the Z lines alone showed some stunted growth and early flowering phenotypes compared to wild-type Col-0 and other mutant variants (Fig. 6b). Importantly, we found that *PR1* expression, a late defence response marker, was significantly upregulated in the Z lines despite the low HopZ5 transgene expression level (Fig. 6c). G plant lines showed *PR1* transcript accumulation levels intermediate between Z lines and wild-type Col-0, while in C plant lines, *PR1* expression was indistinguishable from Col-0. The K plant lines showed only slightly elevated *PR1* transcript accumulation compared to Col-0 (approximately 2-fold), indicating a minor upregulation of defence responses. Next, we assessed whether the transgenic expression of HopZ5 variants could restrict *Pto* DC3000 growth as a result of upregulated defence response. Consistent with *PR1* expression analysis, Z plant lines showed significant reduction in *Pto* DC3000 growth compared to wild-type Col-0 (Fig. 6d). Unexpectedly, the G plant lines did not show significant reduction of *Pto* DC3000 growth compared to Col-0 which could be due to the low expression levels of the G2A variant.

Taken together, the elevated *PR1* expression and reduced *Pto* DC3000 growth relative to wild-type Col-0 in HopZ5 Z lines (Fig. 6c,d), coupled to upregulation of ETI marker genes in response to HopZ5 delivery by *Pto* DC3000 (Supplementary Fig. S1) and virulent pathogen growth restriction (Fig. 1c) suggest that HopZ5 triggers defence that is not associated with HR in Col-0.

## Discussion

We have identified HopZ5 from *P*. *syringae* pv. *actinidiae* NZV-13 as an avirulence effector in Arabidopsis accessions Ct-1 and Col-0. HopZ5 delivery by *Pseudomonas* T3S resulted in HR and bacterial growth restriction in Ct-1. Interestingly, in Col-0, neither HR nor elevated electrolyte leakage was observed but *Pto* DC3000 growth was significantly restricted in the presence of HopZ5. Enhanced defence marker gene expression when HopZ5 was delivered from *Pseudomonas* or transgenically expressed in Col-0 indicates the presence of a functional recognition system in Col-0. The difference between Pf0-1(T3S) and *Pto* DC3000 delivery of HopZ5 is unknown but likely to be an issue of delivery efficiency since in our Pf0-1(T3S) delivery experiments defence marker gene upregulation in Col-0 was poor even for the positive control AvrRpm1 (Fig. 1d). In addition, acetyltransferase activity and plasma membrane localization are required for HopZ5-triggered immunity in both Arabidopsis and *Nicotiana benthamiana*.

Separation of HR from pathogen growth restriction was demonstrated in *ndr1* mutant plants that showed HR in response to *Pto* DC3000 delivering AvrRpm1, AvrB or AvrPphB but lost the ability to restrict growth of these strains^44^. Similarly, Turnip crinkle virus (TCV)-triggered HR is mediated by the CNL gene *HRT* while the full disease resistance is mediated in conjunction with a second recessive determinant *rrt* ^45^. Moreover, TCV resistance has a non-canonical requirement for *EDS1* as well as *RAR1* and *SGT1* but these genes are not required for HR^46^. The converse, HR-independent resistance, was recently shown in Arabidopsis metacaspase mutants, *mc1* and *mc2*. *mc1* and *mc2* mutants show reduced and increased cell death responses to *Pto* DC3000 delivering AvrRpm1, respectively, but have no effect on bacterial growth^47^. Similarly, immunity triggered by unrelated bacterial type III effectors HopA1 (previously HopPsyA) and AvrRps4 specifically in the Col-0 accession are HR-independent^48, 49^. The locus that confers suppression of AvrRps4- or HopA1-triggered HR in Col-0, *HR REGULATOR IN EDS1 PATHWAY* (*HED1*), was previously mapped to the bottom arm of chromosome 5 in Arabidopsis but the exact genetic determinant of this phenotype remains to be elucidated^48^. Furthermore, Heidrich and colleagues have elegantly demonstrated that the cell compartment-specific localization of EDS1 is linked to pathogen growth restriction (nucleus alone) or HR (nucleo-cytoplasmic) in response to *Pto* DC3000 carrying *avrRps4* ^50^.

Yet another example of HR uncoupled from defence is the *defense*, *no death1* (*dnd1*) mutant that shows constitutively elevated defence responses but is unable to mount HR in response to avirulent bacterial strains^51^. *dnd1* carries a null mutation in a cyclic nucleotide-gated ion channel *AtCNGC2* predicted to be involved in Ca^2+^ signalling during defence responses^52^. Interestingly, a similar gain of constitutive defence phenotype was observed in *dnd2*/*AtCNGC4* and *cpr22*/*AtCNGC11*-*AtCNGC12* although the latter is able to show HR^52–54^. The involvement of different *AtCNGCs* in HR-independent defence suggests that they are negative regulators of defence. Notably, it is possible due to its membrane-localization that HopZ5 targets an ion channel in Col-0 triggering HR-independent immunity.

The closest homolog of HopZ5 is AvrBsT from *X*. *campestris* pv. *vesicatoria* (*Xcv*; also called *X*. *euvesicatoria*), sharing 58% amino acid identity. AvrBsT triggers HR and immunity (as measured by virulent bacterial growth) in Arabidopsis accession Pitzal-0 (Pi-0) but not in Col-0 and Landsberg *erecta*-0 (L*er*-0)^55^. Notably, Pi-0 was the only accession out of 71 total tested that showed AvrBsT-triggered HR. Suppression of AvrBsT-triggered immunity/HR is conferred by the *SUPPRESSOR OF AVRBST ELICITED RESISTANCE1* (*SOBER1*) gene encoding a carboxylesterase. In contrast to AvrBsT, Col-0 showed disease resistance to virulent bacterial pathogen carrying *hopZ5* (Figs 1c and 6). Thus, it is likely that the genetic basis of suppression of HopZ5-triggered HR is different from AvrBsT. However, it remains to be seen if *SOBER1* plays a role in HopZ5-mediated immunity in Col-0.

The separation of HR incidence from immunity was originally ascribed to a matter of amplitude, particularly because ETI was perceived as an amplified PTI response^56^. However, as in the aforementioned examples, the resolution of HR from immunity can be complex. In the case of HopZ5, it is conceivable that a dominant suppressor or negative regulator of HR may exist in Col-0. This suppressor would specifically interfere with HR signalling triggered by HopZ5 or reduce the general immunity level below the threshold that is required for HR development but not immunity. Further investigation into the dynamics behind HR-associated and HR-independent HopZ5-triggered immunity could serve to illustrate a larger role of HR in relation to immunity. Indeed, the observation that while *NDR1*- and *EDS1*-silencing did not affect HCD in *N*. *benthamiana*, the loss of either of these genes in Arabidopsis affected immunity against HopZ5, is suggestive of a differentiation between HR (or HCD) from growth restriction. Identification of the putative suppressor of HR in Col-0 plants will enable us to elucidate the point where HR and immunity diverge for HopZ5.

The YopJ family members are present in various plant pathogenic bacteria including *Pseudomonas*, *Xanthomonas*, *Acidovorax* and *Ralstonia* (Supplementary Fig. S2)^19^. *Xcv*, the causal agent of tomato bacterial spot disease, carries four different members of the YopJ family, namely *avrXv4*, *avrRxv*, *avrBsT* and *xopJ* ^57–59^. AvrXv4 is a cytoplasmic effector with small ubiquitin modifier (SUMO)-protease activity that modifies a large number of proteins *in planta* and triggers defence responses in wild tomato relative *Solanum pennellii* and *N*. *benthamiana* ^60^. AvrRxv recruits a plant 14-3-3 protein for its virulence and avirulence activities^61^. AvrBsT, originally shown to trigger disease resistance in pepper plants^62^, is a cytoplasm-localized acetyltransferase that binds to and suppresses cytoplasmic SNF1-related kinase (SnRK1) that is required for AvrBs1-triggered HR^63^. Furthermore, AvrBsT acetylates microtubule-associated proteins to disrupt the microtubule network that is required for plant defence^41^. XopJ, much like its closest homolog, HopZ4, binds and proteolytically degrades proteasomal subunit RPT6 to suppress plant immunity at the plant cell membrane^64^. *R*. *solanacearum* GMI1000 carries three YopJ family members *avrA*, *popP1* and *popP2*. AvrA and PopP1 trigger acetyltransferase activity-dependent HR in *N*. *tabacum* and *N*. *glutinosa*, respectively, that is involved in restriction of host range^28, 65^. The HopZ family of effectors are found in many *Pseudomonas syringae* strains. HopZ1a was shown to target both, JAZ repressors affecting salicylic acid-mediated resistance via activation of jasmonate signalling, and tubulin that results in disruption of the microtubule network and associated protein secretion and defence at the cell wall^22, 24^. HopZ2 from pea pathogen *P*. *syringae* pv. *pisi* 895 A physically associates with an Arabidopsis transmembrane domain containing protein MILDEW RESISTANCE LOCUS2 (MLO2) that is required for resistance against necrotrophic pathogens *Botrytis cinerea* and *Magnaporthe oryzae* ^66^. Cytoplasmic HopZ3 from bean pathogen *P*. *syringae* pv. *syringae* B728a has recently been shown to target Arabidopsis RIN4, various receptor-like cytoplasmic kinases (RLCKs) and the MAP kinase MPK4, all involved in defence signalling *in planta* ^67^. HopZ4 from cucumber pathogen *P*. *syringae* pv. *lachrymans* MAFF301315 binds to proteasomal subunit RPT6 and inhibits proteasome activity that is required for disease resistance^68^. The majority of HopZ effectors have been shown to be localized at the plant cell membrane, likely via myristoylation, and their subcellular localization is required for function^42, 68^. Based on these findings and our results (Figs 3 and 4), it is expected that HopZ5 targets a plasma membrane-localized defence component(s).

Plant resistance against YopJ family members has been genetically characterized so far for HopZ1a, AvrBsT and PopP2. HopZ1a targets pseudokinase ZED1 and this event is recognized by ZAR1^26, 27^. PopP2 directly acetylates WRKY DNA-binding domain of RRS1 and activates RPS4-dependent immunity^28–30^. AvrBsT-mediated immunity in pepper involves both CaSGT1 and CaPIK1^69^. CaPIK1 is a pepper receptor-like cytoplasmic kinase that forms a complex with CaSGT1 and triggers phosphorylation of CaSGT1, subsequent monomerization and nuclear localization. AvrBsT appears to interact with this complex and interferes with this process, leading to a cell death response in an AvrBsT catalytic activity-independent manner but dependent on CaPIK1 phosphorylation of CaSGT1 through specific accumulation of AvrBsT-CaSGT1 in the cytoplasm^69^. Furthermore, two other interactors have been found to contribute to the cell death response triggered by AvrBsT in pepper, CaHSP70a and CaALDH1^70, 71^. As yet it remains unclear how these multiple targets integrate to trigger a cell death response to AvrBsT.

As mentioned previously, few of the characterized homologs of HopZ5 share an *in planta* target while some have a large number of targets that could all additively contribute to its virulence function^67^. Additionally, distinct NLRs recognize corresponding YopJ family T3Es to activate plant immunity. However, certain cues can be taken from previous studies on YopJ family T3Es to predict circumstances for HopZ5. Based on these findings, we hypothesize that HopZ5 could target a host protein(s) that has not been implicated in other HopZ effector-triggered immunity or susceptibility studies. Furthermore, we predict that the *in planta* target and the corresponding NLR of HopZ5 are likely to be plasma membrane localized.

A lysine residue that was shown to be autoacetylated in PopP2 is conserved among the majority of the YopJ family of effectors. In the case of PopP2, the autoacetylated lysine is required for its avirulence activity^18^. However, it was shown that HopZ1a does not require this conserved lysine for its function in a native promoter context^21, 24, 72^. Some members of the YopJ family of effectors were shown to acetylate serine and threonine residues, including YopJ^17^ and HopZ1a^21^. We have shown here that the conserved lysine residue (K278) is required for HopZ5 autoacetylation of lysine residues *in planta* (Fig. 3c). Lysine 278 of HopZ5 seems to either be the only lysine residue that is autoacetylated since the mutation of lysine 278 to arginine (K278R) abolished HopZ5 lysine autoacetylation, or is required for autoacetylation activity. Since the antibody (α-AcK) we used for testing HopZ5 autoacetylation did not allow us to detect non-lysine acetylation, additional autoacetylation of other residues in HopZ5 may still occur in both C218A and K278R variants. The crystal structure of HopZ1a revealed that the analogous residue to K278 in HopZ5, K211 in HopZ1a, is involved in coordination of the plant-derived cofactor IP6^20^. This suggests that HopZ5 K278 could also be involved in coordination of IP6 and thus loss of this residue in the K278R variant results in loss of acetylation activity. Further analysis of HopZ5 autoacetylation and *in planta* target(s) will help unveil the mechanisms involved here. Nevertheless, it is conceivable that K278 could be the only lysine residue critical for HopZ5 avirulence. However, agroinfiltration of K278R variant of HopZ5 in *Nicotiana* spp. could still induce delayed HCD. This partial loss of avirulence activity of the K278R variant of HopZ5 in *Nicotiana* spp. suggests that the requirement of K278 for HopZ5 function might be dose-dependent.

We also observed a similar partial loss of function of the G2A variant which lost plasma membrane localization, presumably through lack of myristoylation (Fig. 3, Supplementary Table 1). This partial loss of function could be due to the cytoplasm-localized HopZ5 G2A variant that could still interact with its target protein and induce HCD. Identification of the host *in planta* target(s) of HopZ5 will be essential in better understanding the requirement for subcellular localization in initiation of HopZ5-triggered immunity in the future.

We show here that *SGT1* is required for HopZ5-mediated HCD in *N*. *benthamiana*. Analysis of the genetic requirement for AvrBsT-triggered immunity in Arabidopsis indicated that *NDR1* (fully), *EDS1* (partially), and *SID2* (partially) were required^55^. A similar study of ZAR1-mediated immunity against HopZ1a in Arabidopsis could not identify requirement for *SID2*, *NDR1*, *EDS1* or *SGT1* ^26^. HopZ5-triggered immunity required *SID2*, *NDR1* and *EDS1*. It must be noted, however, that due to the weak immunity triggered by HopZ5 in the Col-0 background, the full requirements for *SID2* and *EDS1* seen in our experiments may mirror the partial requirements for these two genes for AvrBsT-triggered immunity. Despite this, our results strongly suggest that the recognition of HopZ5 (and possibly AvrBsT) is likely mediated by an as yet uncharacterized NLR. The identification that both *NDR1* and *EDS1* are required for immunity against HopZ5 in Arabidopsis was initially surprising and appeared to contradict our expectations from observations of HCD development in *N*. *benthamiana*. However, due to the uncoupling of HR (or HCD) from immunity in Col-0 plants, we were able to explain the requirement for both *NDR1* and *EDS1* in immunity mounted in response to HopZ5 irrespective of the requirement for HCD (or HR).

Nevertheless, that HopZ5 does not require *NDR1* nor *EDS1* to activate HCD in *N*. *benthamiana*, can be explained by a redundancy between these two canonical pathways in *N*. *benthamiana*, or that the EDS1 or NDR1 levels that do accumulate in the silenced plants are not rate-limiting. Redundancy between *EDS1* and *NDR1* occurs in Arabidopsis *RPP7-* and *RPP8*-mediated immunity against the oomycete pathogen *Hyaloperonospora arabidopsidis* that was not abolished in the absence of *EDS1* or *NDR1* alone but was attenuated in the *eds1 ndr1* double mutant^73^. Therefore, it is plausible that EDS1 and NDR1 play a functionally redundant role in HopZ5-triggered immunity. Alternatively, HopZ5-triggered immunity may require an as yet undiscovered signalling component of ETI.

In addition to *SGT1*, a non-canonical CNL, *NRG1*, is a required genetic component for immunity triggered by the TNL, *N*, against tobacco mosaic virus in *Nicotiana* ^74^. *NRG1* and its homologs, including Arabidopsis *ADR1*, are conserved across all plant families^75^. Interestingly, *ADR1* and *ADR1-L2* were shown to be critical for several CNLs and TNLs^76, 77^. Consequently, it has been suggested that *NRG1/ADR1*-type helper NLRs could be critical downstream genetic components of a large number of canonical NLR-mediated resistances^75–77^. Any involvement of *NRG1* or *ADR1* in HopZ5-triggered immunity in *Nicotiana* or Arabidopsis, respectively, remains to be explored.

Elucidation of the genetic components required for HopZ5-triggered immunity is of particular interest in the interaction between *Psa* and kiwifruit given that HopZ5 is unique to the global outbreak strains of *Psa*. The gain of effectors HopZ5 and HopH1 was proposed as a transposon-mediated lateral transfer event^14^. HopZ5 is unique to *Psa*, while HopH1 is present in multiple pathogenic *Pseudomonas* strains and is believed to be a helper protein^78^. We do not yet know whether this lateral transfer event played a significant role in the rapid global spread of this disease, but if it did this research may help us develop potent options for application of resistance to *Psa*.

## Methods

### Construction of broad-host range vector

The multiple cloning site (MCS) of the broad host range vector, pBBR1MCS-5^79^, was modified by inserting a fusion construct containing 128 bp upstream sequence from the start codon of *avrRps4* (*avrRps4* promoter) and 596 bp of the golden gate cloning system compatible *lacZ* gene containing flanking *Bsa*I restriction enzyme sites from binary vector pICH86988. This fusion was generated by standard PCR using primers 5′-CGCAGATCTTTCCCCGAAGATTAGGAACT-3′ and 5′-GCCAGCTGCGGTCTCCCATTGGGAAGCCTCTTTGTCAAAG-3′ for the *avrRps4* promoter and 5′-CTTTGACAAAGAGGCTTCCCAATGGGAGACCGCAGCTG-3′ and 5′-TATCGATAAGCTGAGACCGTCACAG-3′ for *lacZ* followed by overlap PCR to create a DNA fragment containing 5′-*Bgl*II-*avrRps4*_*pro*_-*lacZ*-*Cla*I-3′. The amplified DNA fragment was digested with *Bgl*II and *Cla*I, purified from a 1.0% agarose gel, and ligated into *Bgl*II- and *Cla*I-treated pBBR1MCS-5. The sequence of the final pBBR1MCS-5:*avrRps4*_*pro*_ construct was confirmed by sequencing with M13F and M13R primers.

### Bacterial type-III effector library construction

Type-III secreted effectors were predicted from the genomic sequence of *Pseudomonas syringae* pv. *actinidiae* NZV-13 by homology to known *Pseudomonas* effectors and annotated using Geneious software [Biomatters, NZ]. All predicted effectors with or without a discernible Hrp box were cloned using the golden gate cloning system^80^ into a binary vector (pICH86988) under the control of a 35 S CaMV promoter and TMV Ω leader for transient expression screens or into the broad host-range vector (pBBR1MCS-5:*avrRps* *4*_*pro*_) for type-III secretion system delivery.

### Bacterial strains

Pseudomonas *syringae* pv. *actinidiae* NZV-13 (ICMP18884) was obtained from Plant & Food Research (Mt Albert, NZ). Cloning of broad-host range and binary vectors was carried out in *Escherichia coli* DH5α. Bacterial type-III effector delivery from the broad host-range vector was achieved using *Pseudomonas fluorescens* Pf0-1 carrying pLN18 (Pf0-1(T3S)). Bacterial pathogen growth curves were carried out using *P*. *syringae* pv. *tomato* DC3000 (NCPPB4369). Transient expression assays in *Nicotiana tabacum* or *Nicotiana benthamiana* was achieved using *Agrobacterium tumefaciens* AGL1. Bacteria were grown on agar solidified L-medium (*E*. *coli* or *Agrobacterium*) or King’s B medium (*Pseudomonas*), with antibiotics appropriate for genomic resistance and/or plasmid vector carried. Plasmids were mobilized from *E*. *coli* DH5α to *Pf* Pf0-1(T3S) or *Pto* DC3000 strains by triparental mating using *E*. *coli* HB101 (pRK2013) as a helper strain.

### Plant material

Arabidopsis accessions used in this research were obtained from The Sainsbury Laboratory (Norwich, UK) or Plant & Food Research (Mt Albert, NZ). Seeds were sown on Dalton’s Premium Seed raising mix [Fruitfed, NZ] mixed with coarse grain vermiculite, transferred to individual cell trays two weeks after germination and grown to 4–5 weeks old before use. *N. benthamiana* and *N. tabacum* Wisconsin 38 seeds were sown on Dalton’s Premium Seed raising mix [Fruitfed, NZ], transferred to individual pots one week after germination and grown for 4–6 weeks before use. Plants were grown in short day conditions with 11 hours light at 22 °C.

### Bacterial infiltrations for HR assays in Arabidopsis

*Psa* or *Pf* Pf0-1(T3S) were streaked from glycerol stocks onto King’s B plates with antibiotic selection and grown for two days at 28 °C. Bacteria were then harvested from plates, resuspended in 10 mM MgCl_2_ and diluted to OD_600_ = 0.2 (1 × 10^8^ CFU/mL) for HR assays. Infiltrations were carried out on fully expanded leaves of 4- to 5-week-old Arabidopsis with a blunt 1 mL syringe. HR was assayed visually at 18–22 hours post infiltration (hpi).

### Electrolyte leakage assays

*Pf* Pf0-1(T3S) strains were grown and infiltrated as for HR assays. Leaf discs were taken with a cork borer from infiltrated leaves when dry, washed in deionized water for half an hour before being floated on deionized water (n = 4). Electrolyte leakage was measured on a Horiba B-173 Twin Cond conductivity meter at indicated time points.

### *N*. *benthamiana* electrolyte leakage

Electrolyte leakage measurements were carried out as described previously^81^. Briefly, Agro-infiltration was carried out as described above and two leaf discs taken per sample (n = 6) at 0 and 2 dpi, floated on 2 mL deionized water with shaking at 160 rpm for 2 hours. Leaf discs were removed before electrolyte measurements were taken.

### *In planta* bacterial growth assays

*Pto* DC3000 strains were streaked and resuspended as for HR assays and diluted to OD_600_ = 0.001 (5 × 10^5^ CFU/mL) before blunt syringe infiltrations into fully expanded leaves of 4- to 5-week-old Arabidopsis plants. At 4 dpi, leaf discs were taken and ground in sterile 10 mM MgCl_2_. Each sample was then serially diluted in sterile 10 mM MgCl_2_ and 20 μL spots of each sample (n = 6) and dilution were plated on King’s B plates with appropriate antibiotics. After 2 days incubation at 28 °C the CFU were counted for the least dilute sample possible.

### *Agrobacterium tumefaciens* transient infiltration

*Agrobacterium tumefaciens* AGL1 strains were streaked onto L-medium plates from glycerol stocks and grown for two days at 28 °C and further inoculated in liquid L-medium with antibiotics and grown overnight before centrifugation and resuspension in Agro-infiltration buffer (10 mM MgCl_2_, 10 mM MES pH 5,6). Suspensions were adjusted to OD_600_ = 0.4 for blunt syringe infiltrations into *N*. *benthamiana* or *N*. *tabacum* leaves. Hypersensitive response-like cell death was assayed at 2–3 days post infiltration (dpi) for tobacco and 3–4 dpi for *N*. *benthamiana*.

### Gene expression by qPCR

Total RNA was extracted from untreated, MgCl_2_ mock-treated or *Pf* Pf0-1(T3S)-infiltrated or *Pto* DC3000-infiltrated leaves from 4- to 6-week-old Arabidopsis plants via Tri-Reagent [Sigma Aldrich, NZ] and BCP [Sigma Aldrich, NZ] extraction. cDNA was synthesized from RNA using the Maxima First strand synthesis kit following manufacturer’s instructions [Thermo Fisher, NZ]. Quantitative PCR was carried out on a Roche Lightcycler™ LCII using SYBR Green mastermix [Thermo Fisher, NZ]. Primers used for qPCR are listed in Supplementary Table 2.

### SDS-PAGE and Western blot

*N. benthamiana* leaves were infiltrated with *A. tumefaciens AGL1* carrying binary vectors for tagged proteins. Leaf tissue was harvested at 2 dpi and snap frozen before grinding and total protein extraction. Samples were immunoprecipitated (IP) using anti-FLAG affinity gel [Sigma Aldrich, NZ]. Total protein and IP samples were boiled in loading buffer containing DTT [Sigma Aldrich, NZ] to denature proteins. SDS-polyacrylamide gel electrophoresis was performed, subsequently blotted onto PVDF membranes [Sigma Aldrich, NZ] and probed with horseradish peroxidase-conjugated antibody against the epitope tag (α-FLAG [Sigma Aldrich, NZ] or α-GFP [Santa Cruz Biotechnology, USA]) or against acetylation (α-AcK) [Cell Signaling, USA]. Visualization was achieved using Pierce Pico and Femto reagents [Thermo Fischer, NZ]. Protein loading was visualized using Ponceau staining [Thermo Fischer, NZ].

### Virus-induced gene silencing

Two-week-old *N*. *benthamiana* seedlings were infiltrated with pTRV1 and pTRV2 (carrying the target gene sequence) at total OD_600_ = 1 (Mixture of OD_600_ = 0.5 of each pTRV1 and pTRV2), into 2 cotyledons. Infiltrated plants were grown 4–5 weeks before infiltration with *A*. *tumefaciens* AGL1 carrying YFP-tagged *hopZ5*. Samples were also taken from infiltrated plants to confirm silencing by RNA extraction and qPCR.

### Confocal microscopy

*SGT1*-silenced *N*. *benthamiana* plants were transiently infiltrated with *A*. *tumefaciens* AGL1 carrying YFP-tagged HopZ5 variants. Leaf discs were taken at 2 dpi from randomly selected infiltrated area, avoiding leaf veins, and mounted in water for confocal laser scanning microscopy. A Carl Zeiss LSM700 with excitation at 488 nm with a 20 mW Argon laser with emission filters set between 500 and 530 nm was used for imaging.

### Stable transgenic line generation

*A. tumefaciens AGL1* carrying C-terminally YFP-tagged HopZ5 constructs in binary vectors were transformed into Arabidopsis plants (Col-0) by floral dip transformation as described previously^82^. Transgenic plants were selected on agar-solidified Murashige and Skoog (MS) [Duchefa, Total Labs, NZ] plates containing 50 μg/mL kanamycin and confirmed by qPCR for transgene expression. T2 seeds harvested from each confirmed T1 parent line were used for experiments after selection on agar-solidified MS medium supplemented with kanamycin.

## Electronic supplementary material

Supplementary Information
