# Supplementary material for: A bacterial acetyltransferase triggers immunity in Arabidopsis thaliana independent of hypersensitive response
Source: Sci Rep. 2017 Jun 15;7:3557. doi: 10.1038/s41598-017-03704-x (PMC5472582; doi:10.1038/s41598-017-03704-x)
Supplement: Supplementary file 1 — Supplementary Information [file 41598_2017_3704_MOESM1_ESM.pdf]

Supplementary Information:

A bacterial acetyltransferase triggers immunity in *Arabidopsis thaliana* independent of hypersensitive response

Jay Jayaraman[1,5,a], Sera Choi[1,5,a], Maxim Prokchorchik[1,5], Du Seok Choi[5], Amandine Spiandore[1], Erik H. Rikkerink[2], Matthew D. Templeton[2,3], Cécile Segonzac[4,5] and Kee Hoon Sohn[1,5,6,*]

[1]Bioprotection Research Centre, Institute of Agriculture and Environment, Massey University, Palmerston North 4474, New Zealand
[2] New Zealand Institute for Plant & Food Research Limited (PFR), Private Bag 92169, Auckland, New Zealand
[3]School of Biological Sciences, University of Auckland, Private Bag 92019, Auckland, New Zealand
[4]Department of Plant Science, Plant Genomics and Breeding Institute, College of Agriculture and Life Sciences, Seoul National University, Seoul 08826, South Korea
[5]Department of Life Sciences and [6]School of Interdisciplinary Bioscience and Bioengineering, Pohang University of Science and Technology, Pohang 37673, South Korea
[a]These authors contributed equally to this research
[*]Corresponding author. Email: khsohn@postech.ac.kr

Supplementary Figure S1

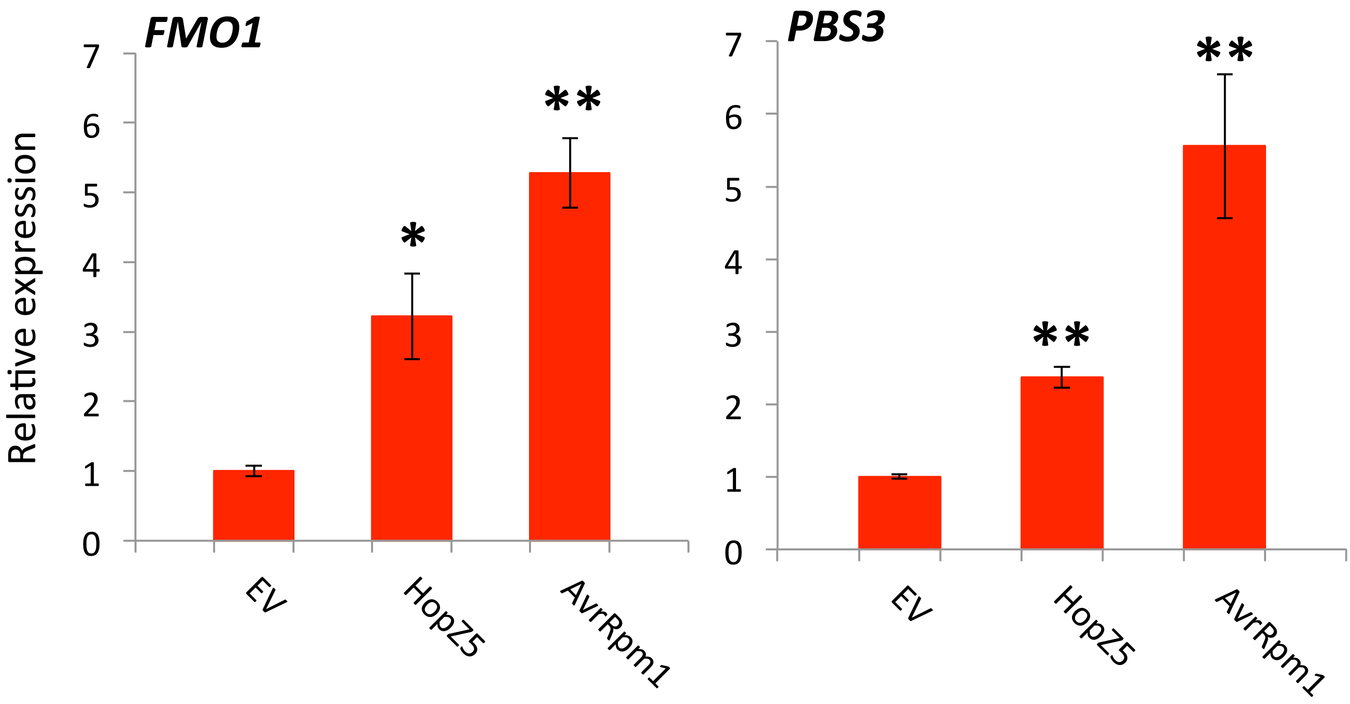

Supplementary Figure S1. ***Pto*** **DC3000-delivered HopZ5 triggers ETI in** ***Arabidopsis thaliana*** **Col-0.** Defence gene expression in Arabidopsis accession Col-0 in response to EV, HopZ5-HA or AvrRpm1. *Pto DC3000* (2×10^7 CFU/mL) carrying EV, *hopZ5*-HA or *avrRpm1* was blunt syringe-infiltrated into leaves, samples taken at 8 hours post infiltration and defence gene expression determined from extracted RNA by quantitative polymerase chain reaction (qPCR). Expression for each defence gene is relative to internal *EF1α* expression and defence gene expression for EV samples indicated. Error bars indicate standard error from three technical replicates. Asterisks indicate results of Student's t-test between selected sample and EV; * ($P < 0.05$), ** ($P < 0.01$). The experiment was conducted twice with similar results.

Supplementary Figure S2

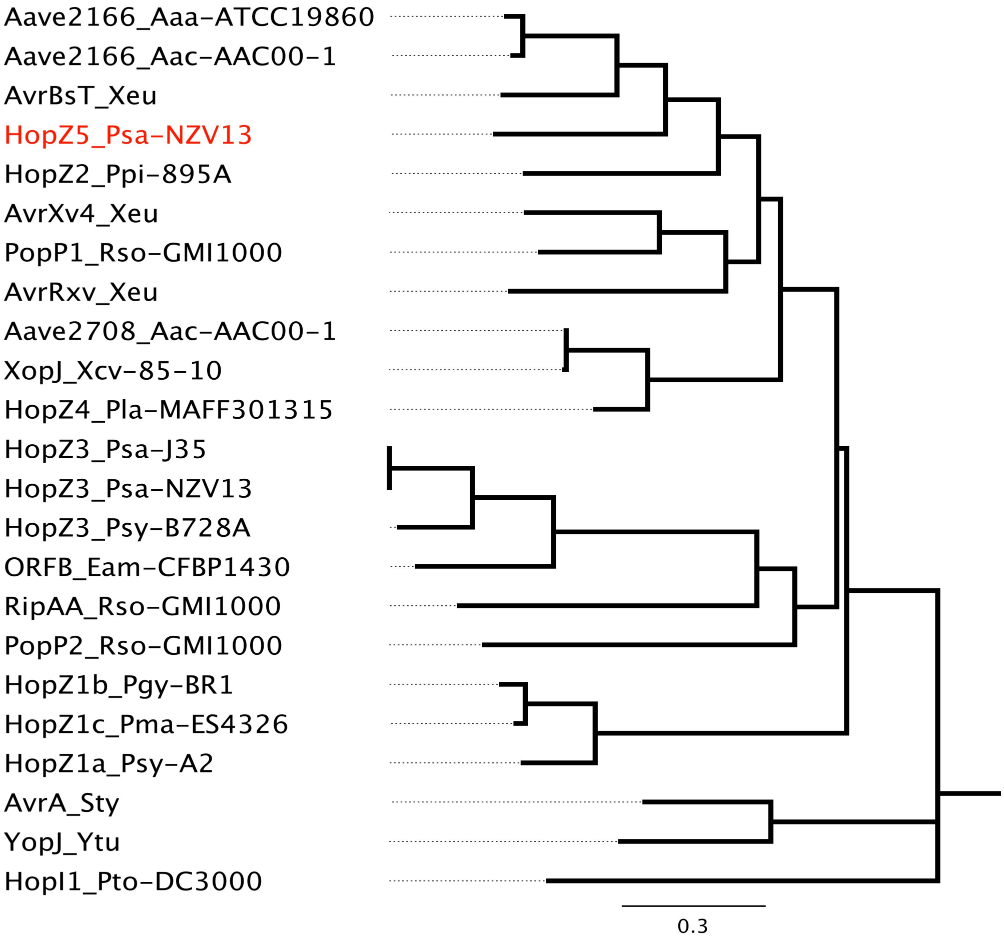

Supplementary Figure S2. HopZ5 is a member of the YopJ family of putative acetyltransferases. HopZ5 (in red) from *Pseudomonas syringae* pv. *actinidiae* NZV-13 is a putative acetyltransferase from the YopJ family with *Xanthomonas* type-III secreted effector AvrBsT as its closest homolog. Jukes Cantor neighbour-joining tree using HopI1 from *Pto* DC3000 as outgroup and root, generated in Geneious software with various YopJ-family members: Aave2166_Aaa-ATCC19860 (*Acidovorax avenae* pv. *avenae* ATCC19860), Aave2166_Aac-AAC00-1 (*Acidovorax citrulli* AAC00-1), AvrBsT_Xeu (*Xanthomonas campestris* pv *euvesicatoria* Bv5-4a), HopZ2_Ppi-895A (*P. syringae* pv. *pisi* 895A), AvrXv4_Xeu (*X. campestris* pv *euvesicatoria* race T3), PopP1_Rso-GMI1000 (*Ralstonia solanacearum* GMI1000), AvrRxv_Xcv (*X. campestris* pv *euvesicatoria* 85-10), Aave2708_Aac-AAC00-1 (*Acidovorax citrulli* AAC00-1), XopJ_Xcv-85-10 (*X. campestris* pv. *vesicatoria* 85-10), HopZ4_Pla-MAFF301315 (*P. syringae* pv. *lachrymans* MAFF301315), HopZ3_Psa-J35 (*P. syringae* pv. *actinidiae* J35), HopZ3_Psa-NZV13 (*P. syringae* pv. *actinidiae* NZV-13), HopZ3_Psy-B728A (*P. syringae* pv. *syringae* B728A), ORFB_Eam-CFBP1430 (*Erwinia amylovora* str. CFBP1430), RipAA_Rso-GMI1000 (*R. solanacearum* GMI1000), PopP2_Rso-GMI1000 (*R. solanacearum* GMI1000), HopZ1b_Pgy-BR1 (*P. syringae* pv. *glycinea* BR1), HopZ1c_Pma-ES4326 (*P. syringae* pv. *maculicola* ES4326), HopZ1a_Psy-A2 (*P. syringae* pv. *syringae* A2), AvrA_Sty (*Salmonella typhimurium* str. *14028s), YopJ_Ytu (Yersinia pseudotuberculosis str.* IP32953). Node length is indicated.

Supplementary Figure S3

Myristoylation site

```
HopZ1a   1 MGNVCVG---GSRMSHQVYSPDRADTPP 25
XopJ     1 MGLCVSKPSVAGSPEHYAAHVAEQATPS 28
HopZ2    1 MGICVSKPSVRHDYNEDYGRNYGADTAQ 28
HopZ5    1 MGLCASKPRTTSGYNTYASYNSGSSTPE 28
```

Catalytic sites

```
PopP2   256 DDGSHTRAADIRKDASG-TSVIVVDPL-------RKEKDESAYVDYADNVNMEFGEHAKCAFIPVDIQKSFFDCRILSLSLAL 330
AvrA    119 SSGIHISVVDFRVM-DGKTSVILFEPA-----ACSAFGPALA-LRTKAALEREQLPDCYFAMVELDIQRSSSECGIFSLALAK 194
YopJ    105 EGGIHFSVIDYKHI-NGKTSLILFEPA-----NFNSMGPAMLAIRTKTAIERYQLPDCHFSMVEMDIQRSSSECGIFSFALAK 181
HopZ1a  146 QTLKHHVMADVRLHQGGAPTIIITEPAVIVG---ARYQQLQRHNLTLEDLSESGVPLSQVAIIETQAQKTSDDCVMYSLNYAI 225
XopJ    169 RDGEHHVAADVRRRPNGEASVIVLEPA-------RLLTFVTGHTQLRRQALSQLGENAKFAFIQVGAQKSAADCLMFDLHFAL 244
PopP1   162 PN-LHHFAADVRHHEDGRTTVIILEPA-----SAGNQENLPGYTELASALRYNLGSQCRMVVIEAEAQKSLSDCVAFALDFAL 238
HopZ2   156 PPSLHHVAVDVRNHSNGQKTLIVLEPITAYKDDVYPPAYLPGYPQLREEVNTRLRGNAKMSVIETDAQRSWHDCVIFSLNFAL 238
AvrBsT  150 PSSMHHAAIDVRFK-DGKRTMLVIEPALAYGMKDGEIKVMAGYETLGKNVQNCLGENGDMAVIQLGAQKSLFDCVIFSLNMAL 231
HopZ5   146 PNSRHHVAIDVRNQ-DDVRTMLIIESALAYGKSNNATGFLPGYLQMHNNVKNYAQDNGRMAVIQLGVQKSKYDCIIFSLNNAL 227
```

Autoacetylation-required site

```
PopP2   373 GAPLVDARMMKHGQAASSVSRYLGNHPEQSTVPVNKR-----NETLGERTTRH 420
AvrA    227 ADRYLPVSFYKHTQGAQRLNEYVEANPAAGSSIVNKK-----NETLYERFDNN 274
YopJ    214 LDPYLPVTFYKHTQGKKRLNEYLNTNPQGVGTVVNKK-----NETIVNRFDNN 261
HopZ1a  279 GADVLPVDFYKHGASLTQAKQLMKRPDGRMAGRVNSE-GHSEAENLVQRNQAF 330
XopJ    290 GEQMLPAIFYKHTHSSGVVEEVDRSQPGSAYTDVSTSGRQQQHESLEQRVQAF 342
PopP1   289 GWGVLPPVFYKHAHSRETLKGVEKRQPGSLETDVSTGRNKDGAESLEERMEAF 341
HopZ2   280 GKQVLPAVFYKHAHSRGTVTAVENAQPHIANDNVSTN-RSSSRETLNERAEAF 331
AvrBsT  272 GDKFLPPIFYKHSHSRGVVGEFISNQPEYAHKNVSTG-RTNPSEDLSERVENF 323
HopZ5   268 GTKILPAVFFKHTHSRSTINDAIEEQPELADRNVSTN-RESAHQTLSERVADF 319
```

Supplementary Figure S3. HopZ5 shares predicted conserved residues with YopJ family members. Critically conserved residues in selected YopJ family members are shown: Putatively myristoylated glycine highlighted in purple, acetyltransferase catalytic core histidine, glutamate and cysteine residues highlighted in orange, and autoacetylation-required lysine residue highlighted in blue.

Supplementary Figure S4

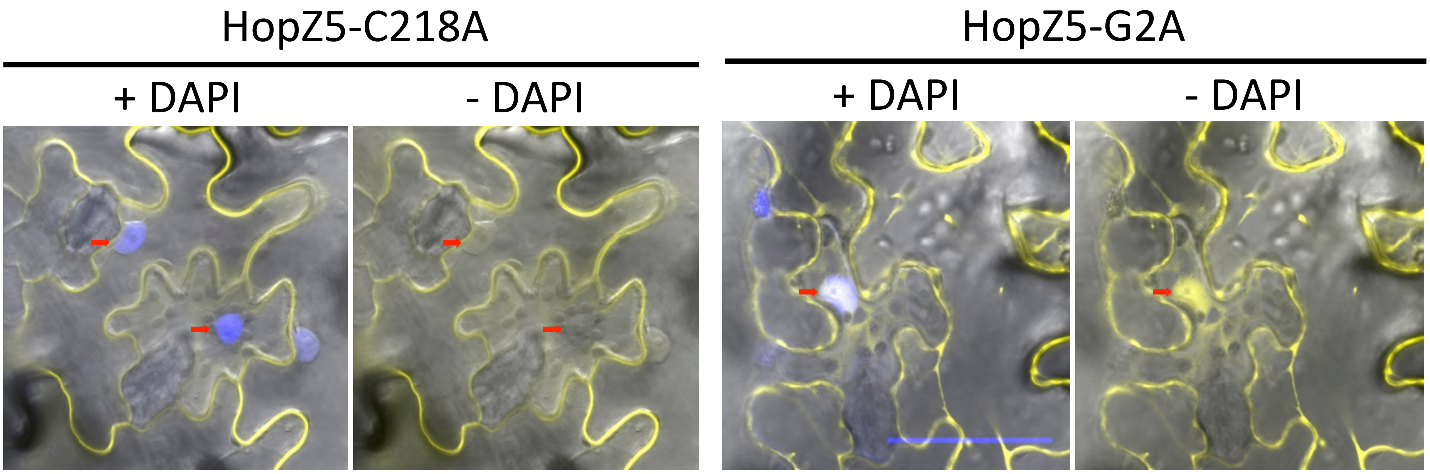

Supplementary Figure S4. The G2A variant of HopZ5 localizes to the plant cell cytoplasm and nucleus. *In planta* subcellular localization of HopZ5 variants was determined in *SGT1*-silenced *N. benthamiana.* Leaves were infiltrated with *Agrobacterium tumefaciens* AGL1 carrying YFP-tagged *hopZ5* (G2A) or *hopZ5* (C218A) under a constitutive CaMV 35S promoter. Leaf discs were taken at 2 days post infiltration, stained with DAPI, and visualized under confocal laser-scanning microscopy for localization of the DAPI signal and YFP tag. All panels are confocal images merged with corresponding bright-field images, with or without DAPI signal included as indicated. Red arrows indicate plant cell nuclei. The blue scale bar in G2A panel represents 50 µm.

Supplementary Figure S5

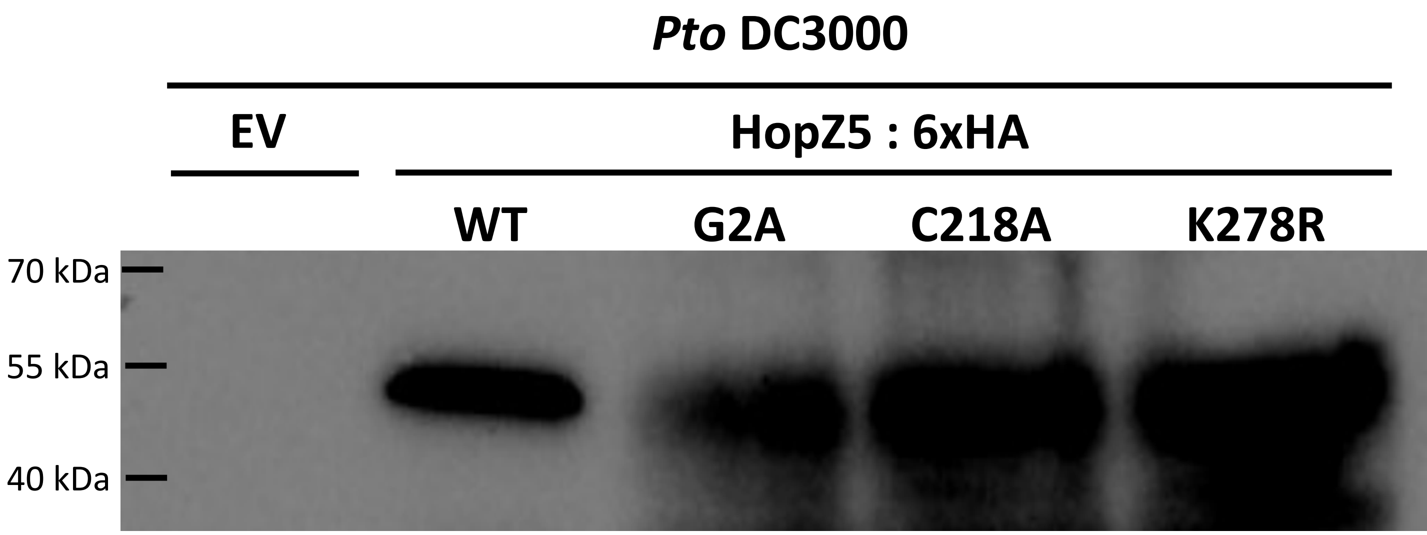

Supplementary Figure S5. HopZ5 variants are unaffected in expression and protein stability in *Pto* DC3000. *Pto* DC3000 carrying *hopZ5* (WT) or with mutated myristoylation (G2A), catalytic core (C218A), or autoacetylation-required (K278R) variants tagged with 6xHA were inoculated at OD_{600}=0.15 and grown for 7 hours on type-III inducing minimal medium (Huynh et al. 1989)*. Cells were harvested by centrifugation, boiled in 1xLeamli buffer and separated by SDS-PAGE for immunoblot using α–HA antibody.

*Huynh, T., Dahlbeck, D. & Staskawicz, B., Bacterial blight of soybean: regulation of a pathogen gene determining host cultivar specificity, *Science* **145**, 1374-1377 (1989)

Supplementary Figure S6

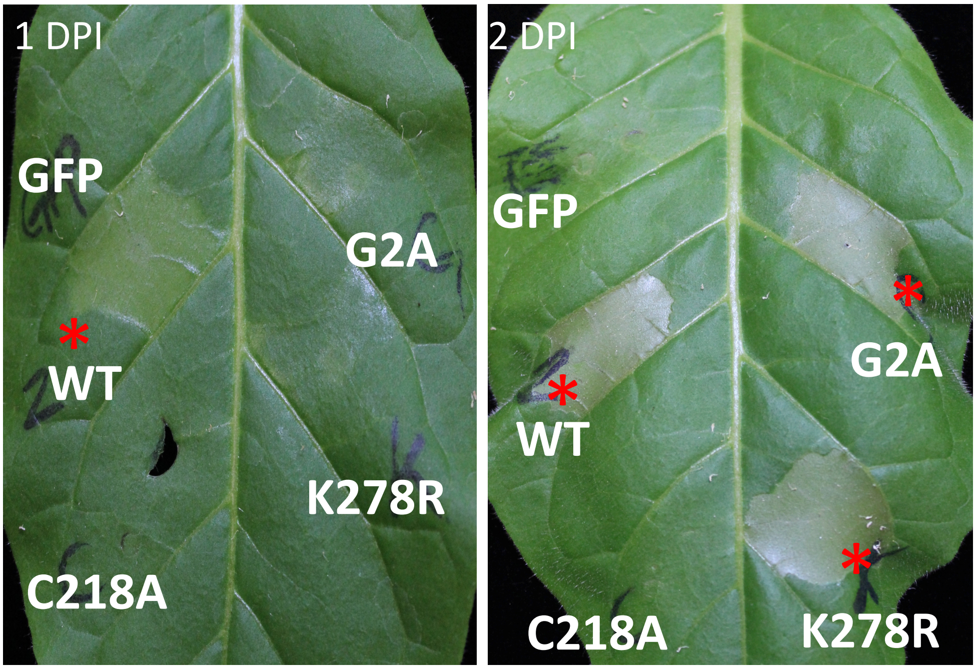

Supplementary Figure S6. HopZ5 triggers a hypersensitive response in *Nicotiana tabacum* and requires G2, C218 and K278 residues. HopZ5 (WT) with mutated myristoylation (G2A), catalytic core (C218A), or autoacetylation (K278R) variants tagged with 3xFLAG were transiently expressed under the constitutive CaMV 35S promoter in *Nicotiana tabacum*. Photographs were taken at 1-2 days post infiltration (dpi). Red asterisks indicate development of hypersensitive response-like cell death symptoms. This experiment was repeated five times with identical results.

Supplementary Figure S7

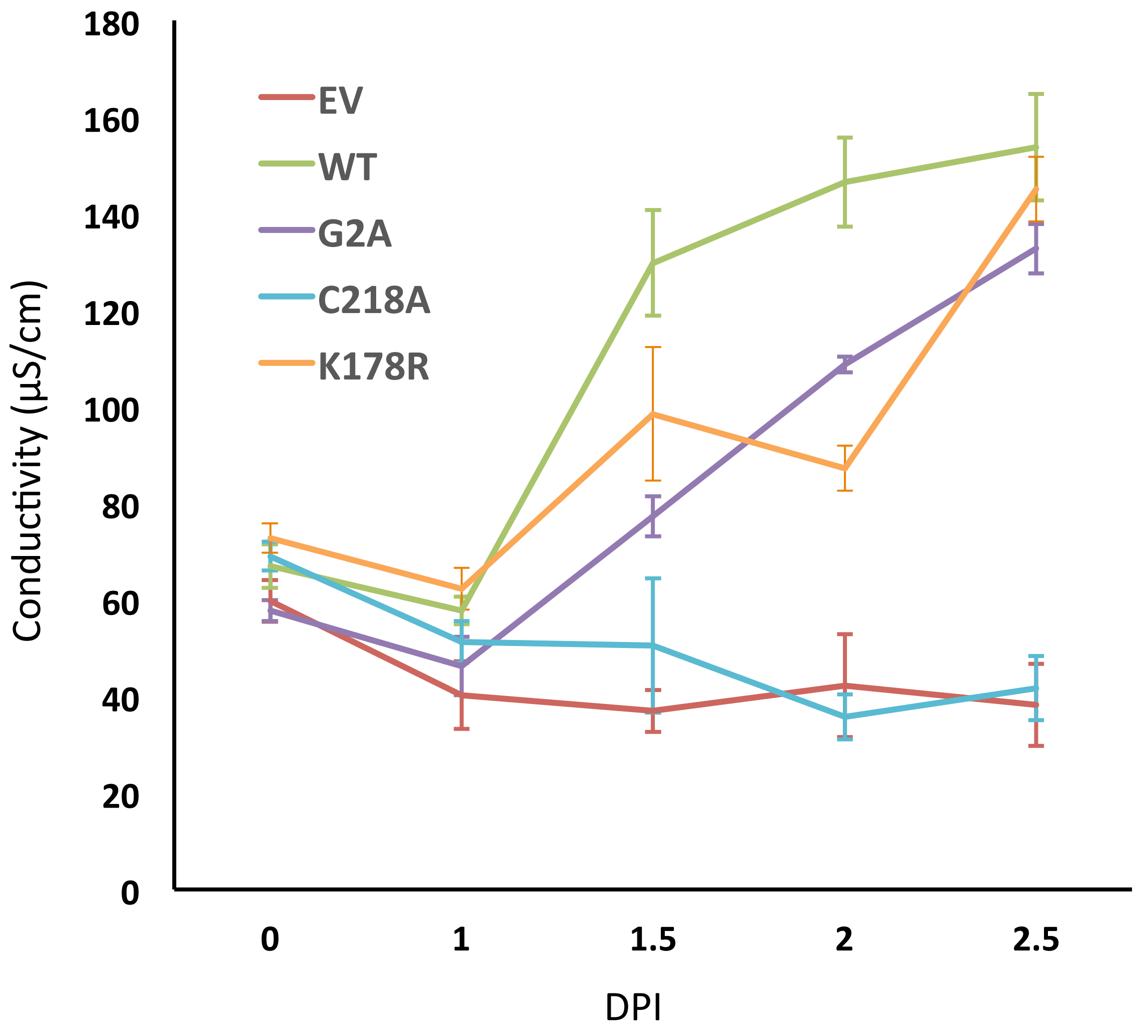

Supplementary Figure S7. HopZ5 recognition in *Nicotiana benthamiana* requires acetyltransferase activity. Electrolyte leakage from *N. benthamiana* leaf discs triggered by *Agrobacterium*-mediated transient expression of HopZ5 or variants was determined at multiple time points (dpi). Infiltrations were carried out by blunt syringe at OD_{600}= 0.4 for all samples as described in Figure 5(b).

Supplementary Table 1. Summary of phenotypes for HopZ5 variants by assay

Assay	WT	G2A	K278R	C218A
HR (*Pseudomonas* delivery)	+++	+++	-	-
Electrolyte leakage (*Pseudomonas* delivery)	+++	++	-	-
Defence gene expression (*Pseudomonas* delivery)	+++	+ / -	-	-
Pto DC3000 growth restriction	+++	+++	+ / -	-
Defence gene expression (Col-0 stable expression)	+++	++	+ / -	-
Pto DC3000 growth restriction (Col-0 stable expression)	+++	-	-	-
HR (*Nicotiana* agroinfiltration)	+++	++	++	-
Electrolyte leakage (*Nicotiana* agroinfiltration)	+++	++	++	-

+++ = strong phenotype with statistically significant difference compared to empty vector control

++ = intermediate phenotype with statistically significant difference compared to empty vector control

+/- = weak phenotype; variable results and occasionally not statistically significant compared to empty vector control

\- = not statistically significant as compared to empty vector control

Supplementary Table 2. Primers used for qPCR of marker genes

Gene	Forward	Reverse
AtEF1α	CAGGCTGATTGTGCTGTTCTTA	GTTGTATCCGACCTTCTTCAGG
AtPBS3	TTCGCTGGCTTGTATAGGATGA	CTGGAAATGTTGAGGTGTCAGC
AtFMO1	TGTGTTTGAAGATGGGACGACA	GTTCGAGCTGCTTTGGACGTAT
AtWRKY51	AGGAAGTATGGCAAGAAATCTG	GAGTGTTGGTTCCAGTTATCAT
hopZ5	TGCATCAAAACCCCGCAC	GTGATATACCCTGACTGCC
AtPR1	ATACACTCTGGTGGGCCTTACG	TACACCTCACTTTGGCACATCC
NbACTIN	GATGAAGATACTCACAGAAAGA	GTGGTTTCATGAATGCCAGCA
NbSGT1	TAATGTGTCATCAGATGCCC	ACTTCTTTCCAGTTTGTCGAC
NbNDR1	TAGTAAAGTGAAAGTGGATGGTTC	GCAATCAACTGAGTCCAACAT
NbEDS1	AACGAGGAAAAGATTGATGGTA	TCCTTTCTTCCCTCAAAACTATC
